# miRNAs: possible regulators of toll like receptors and inflammatory tumor microenvironment in colorectal cancer

**DOI:** 10.1186/s12885-024-12417-0

**Published:** 2024-07-10

**Authors:** Marwa Matboli, Nourhan Hossam, Doaa Farag, Mohamed Hassan, Hanan Shehata, Marwa Aboelhussein, Nahed Ismail, Sanaa Eissa

**Affiliations:** 1 Department of Medical Biochemistry and Molecular Biology, Faculty of Medicine, Ain Shams University, Cairo, Egypt; 2 Department of Pharmaceutical Chemistry, Faculty of Pharmacy, Misr International University, Cairo, Egypt; 3 Department of Biology/Zoology, Biotechnology Program, Faculty of Science, Port Said University, Port Said, Egypt; 4 Zewail City for Science & Technology, Center for Genomics, Helmy Institute for Medical Science, Giza, Egypt; 5 Department of Pathology, University of Illinois at Chicago, Chicago, IL 60612 USA; 6 MASRI Research Institute, Faculty of Medicine, Ain Shams University, Cairo, Egypt

**Keywords:** Colorectal cancer, Toll-like receptor, MicroRNA, Ligand, Mimic, Docking

## Abstract

**Background:**

Colorectal cancer (CRC) is ranked as the third most commonly diagnosed cancer and the third cause of cancer related deaths. CRC is greatly attributed to genetic and epigenetic mutations and immune dysregulation. Tumor aberrant expression of Toll-like Receptors (TLRs) can contribute to tumorigenesis. Recent studies suggested that microRNAs act as direct ligands of TLRs altering their expression and signaling pathways.

**Aim:**

To prove our concept that specific miRNA mimics may act as antagonists of their specific toll like receptors inhibiting their expression that could limit the release of pro-inflammatory and pro-tumorigenic cytokines leading to apoptosis of tumor cells.

**Methods:**

From public microarray databases, we retrieved TLRs and miRNAs related to CRC followed by in silico docking of the selected miRNA ligands into the TLRs. Clinical validation after co-immunoprecipitation of TLRs and their interacting miRNA ligands was done. Expression of TLRs 1, 7,8 was determined by ELISA while miRNAs was measured by RT-qPCR. In addition, microRNA mimics of the down regulated miRNAs were transfected into human CRC cell lines.

**Results:**

Our data demonstrate that TLRs 1, 7, 8 are up regulated in CRC compared to controls. Further, three miRNAs (-122, -29b and -15b) are relatively downregulated, while 4 miRNAs (-202, miRNA-98, -21 and -let7i) are upregulated in CRC patients compared to those with benign tumor and healthy controls. Transfection of down regulated miRNA mimics into CRC cell lines resulted in a significant reduction of the number and viability of cells as well as down regulating the expression of TLRs 1, 7 and 8 with ultimate reduction of downstream effector IL6 protein, suggesting that these miRNAs are negative regulators of carcinogenesis.

**Conclusion:**

MicroRNAs could act as antagonistic ligands of TLRs limiting the inflammatory tumor microenvironment.

**Supplementary Information:**

The online version contains supplementary material available at 10.1186/s12885-024-12417-0.

## Key points

• Specific miRNAs were identified by in silico docking as signaling antagonists of 3 TLRs and were validated in clinical samples.

• TLRs 1, 7, 8 were upregulated while their miRNAs (-122, -29b and -15b)were down regulated in CRC.

• Transfection of downregulated miRNA mimics into CRC cell lines resulted in downregulation of TLRs that was associated with reduction in IL6 protein and tumor growth.

• The present study proposed a novel approach that enables a reliable integration of Toll like receptors and miRNAs in CRC pathogenesis.

**Supplementary Information:**

The online version contains supplementary material available at 10.1186/s12885-024-12417-0.

## Introduction

Colorectal cancer (CRC) is the third most commonly diagnosed cancer worldwide. It represents the third leading cause of cancer related mortality in men and women as well [1]. Many factors are implicated in the pathogenesis of CRC including gene mutations, epigenetic alterations, local inflammatory responses, and immune system deregulation [2]*.* However, it has become increasingly clear that chronic inflammation of the intestine is a major carcinogenic process. The resulting release of inflammatory cytokines and the ensuing immune reaction result in epigenetic changes, recruitment of immune cells as well as pro-tumorigenic signals that contribute to initiation and progression of tumor growth [3].

Toll-like receptors (TLRs) are a diverse family of Pattern Recognition Receptors (PRR) expressed by intestinal epithelial cells, tumor cells and immune cells from both the innate and adaptive arms of the immune system [4]*.* Several studies highlighted the dual role of certain TLRs in cancer progression as well as anti-cancer immune responses. It was found that TLRs on the surface of tumor cells with their ligands can activate subsequent signaling cascades and recruit more immune cells in the tumor microenvironment to enhance inflammation and this TLR-induced inflammation might stimulate tumor growth and spread into the microenvironment [5]*.* On the other hand, aberrant expression of certain TLRs by tumor cells correlated with growth inhibition and antitumor response [6].

MicroRNAs (miRNAs) are a class of small non-coding RNAs with an average 22 nucleotides in length. Although the conventional role of microRNAs is regulation of gene expression, recent studies suggested that microRNAs can act as direct ligands of TLRs [7]*.* For example, miRNA-21 released from cancer cells exosomes was found to bind to TLR7 and ultimately led to TLR-mediated tumor metastasis and growth [8]*.* Also, studies found that cancer cell-derived exosomal miRNAs can bind to TLRs in macrophages and intestinal cells and stimulate NF-κB pathway with subsequent release of the pro-inflammatory and pro-metastatic cytokines IL-6 and TNF-α which stimulate tumor growth and metastasis [9]*.* MicroRNA molecules are now at the center of molecular oncology, with applications for diagnosis and therapy starting to be proposed. The ability of miRNAs to regulate important cellular processes by concurrently regulating multiple targets illustrates their potential as a viable therapeutic tool [10]. miRNA have crucial role in GIT malignancy [11] including CRC [12].

The development of miRNA-based therapy for cancer treatment is based on the premise that aberrantly expressed miRNAs play a crucial role in the development of cancer and therapeutic response to anticancer drugs [13]. Therefore, correcting the miRNA deficiency or restoring the miRNA function could be used as a novel strategy in cancer treatment [14]*.* Based on the fact that some miRNAs have dual role in regulation of gene expression; a)the tumor suppressive role of the selected miRNAs through binding to Argonaute proteins and guide the silencing of target genes; b) on the other hand, they act independently of Argonaute proteins by interacting directly with TLRs as an antagonist thus representing a possible target for cancer treatment [15]. So, we hypothesize that specific miRNA mimics may act as antagonists of their specific toll like receptors inhibiting their expression that could limit the release of pro-inflammatory and pro-tumorigenic cytokines leading to apoptosis of tumor cells.

**Fig. 1:**
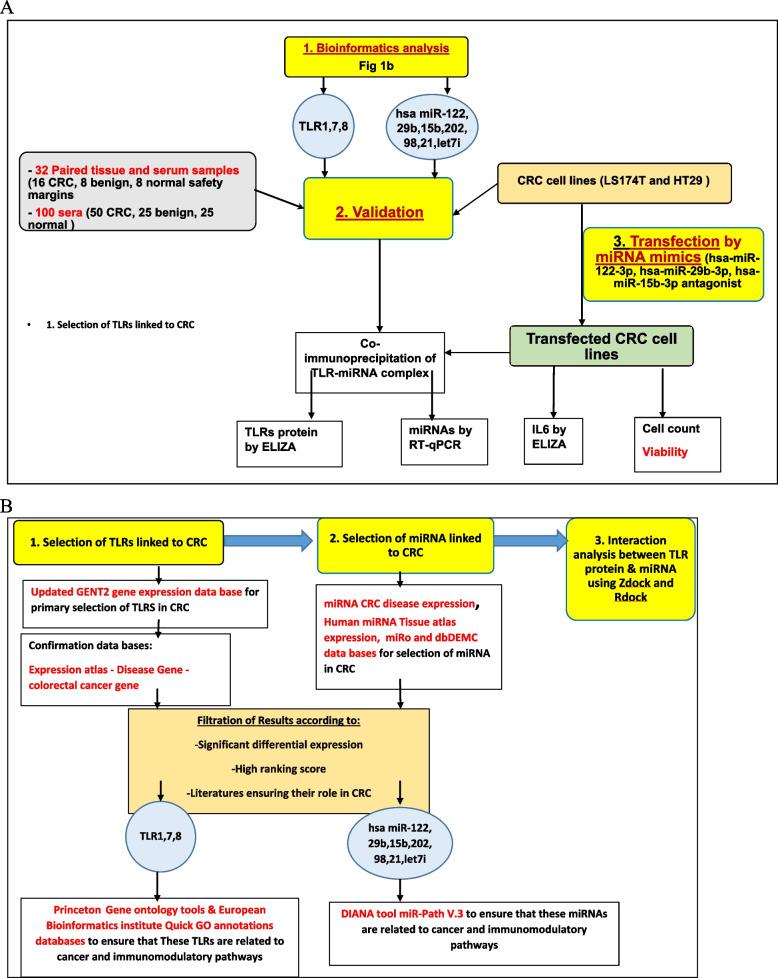
**A** flow chart of the experimental procedures performed to validate the study hypothesis. **B** Rationale for bioinformatics based selection of miRNA-TLR specific to CRC pathogenesis

To prove this hypothesis, we identified a panel of miRNA ligands that interact with specific toll like receptors to regulate their expression and signaling in CRC using bioinformatics methods. Then, we confirmed their presence in patient’s samples and cell lines, lastly, we investigated the efficacy of specific miRNA mimics in CRC growth inhibition A summary of the of the study experimental procedures is presented in (Fig. 1A).

## Materials and methods

### In-silico analysis

Bioinformatics based selection of miRNA-TLR specific to CRC pathogenesis is provided in (Fig. 1b). The COSMIC database v78 (http://cancer.sanger.ac.uk/cosmic) was downloaded and interrogated for *TLR1,7 & 8* mutations. Mutations were counted by cancer type as defined by the filters for tissue type and described in S1&2 Table and figure S1. Public microarray databases were used to examine the expression of candidate TLRs in CRC tissues and normal tissues using updated GENT2 Gene expression database [16] (Available at: http://gent2.appex.kr/gent2/, Accessed on: 18/9/2022). Three TLRs; TLR1, TLR7 and TLR8 were selected based on high ranking score of their differential expression in CRC compared to controls by Two-sample T-test analysis (*p* value < 0.001 and Log2FC = -0.309 for TLR1, *p* value < 0.001 and Log2FC = -0.806 for TLR7 and with *p* value = 0.004 and Log2FC = -0.157 for TLR8. The chosen CRC specific TLR 1,7 and 8 were verified in previous literatures [14] and other databases e g. Expression atlas, Disease Gene and colorectal cancer gene databases to decrease the false discovery rate. Then, gene ontology tracking of the three TLRs was done through Princeton Gene ontology tools database and European Bioinformatics institute Quick GO annotations database [17].

Moreover, seven miRNAs were identified from public databases (Colon cancer microRNA, Human miRNA Tissue atlas expression database, miRo: a miRNA knowledge base and dbDEMC: database of differentially expressed miRNAs in human cancers [18]). Hsa-miR122, hsa-miR29b, hsa-miR 15b, hsa-miR202, hsa-miR 98, hsa-miR 21 and hsa-miR let 7i were selected according to their high ranking score of their differential expression in CRC compared to control. The results were verified in previous literatures [15–21]. Then, pathway enrichment analysis of the seven miRNAs using DIANA tools database was carried out. Lastly, we examined the direct interaction between selected miRNAs and the TLRs via the in silico analysis. Discovery studio 3.5 software (DS 3.5; Accelrys Co. Ltd, San Diego, California, USA) was used for homology modelling and docking of miRNAs into TLRs [19, 20]. ZDOCK predicts all possible binding poses between the miRNA ligand and TL receptor and evaluates each pose using ZDOCK score, an energy-based scoring function which is a combination of pairwise shape complementarity, desolvation and electrostatics [21, 22] (Further details of docking methodology is provided in supplementary Figure S17-23). ZDOCK is a docking program that predicts all possible binding poses in the translational and rotational space between the ligand and receptor and evaluates each pose using ZDOCK score, an energy-based scoring function [23]. ZDOCK generated 60 clusters containing 2000 structures and ranked them according to their ZDOCK score. While, RDOCK is a program designed to refine and re-rank top predictions from ZDOCK using energy minimization algorithm [22].

### Patient population

This study was approved by the Ethical Committee of the Faculty of Medicine, Ain Shams University (FMASU MD 379/2018) and all experimental procedures were in compliance with the Declaration of Helsinki and informed consents were obtained from all study participants. CRC cases were diagnosed according to The American Cancer Society (ACS) guidelines for diagnosis of CRC [24]. Before any intervention such as radiotherapy, chemotherapy or even surgery, 32 paired serum and tissue samples were initially collected from colonoscopy unit at Ain Shams University hospitals in the period from December 2019 till the end of 2021. Samples were classified as 16 CRC, 8 benign colorectal neoplasms and 8 normal samples from patients with normal colon mucosa.

Tissues were stored at -80 °C till further analysis. Then, other 100 sera samples were collected from patients at the oncology Clinic, General Surgery Department, Ain Shams University Hospitals to be added to the previous collected samples from colonoscopy unit. Altogether, serum samples included 50 samples from CRC patients, and 25 from patients with benign colorectal neoplasm. Moreover, 25 sera were obtained from healthy volunteers with matching age and sex to the patients’ groups.

All tumors were generally characterized as primary moderately differentiated adenocarcinomas via colonoscopy. The clinical stages of the CRC patients were determined according to the latest TNM staging system of the American Joint Committee on Cancer (AJCC) [25] where 30%-37% patients were in early stage (stage 0, 1) and 63%-70% patients were in late stage of CRC (stage 2, 3, 4). The clinical data for every patient were available in details. The serum levels of Carbohydrate Antigen (CA19.9) and Carcino-embroyonic Antigen (CEA), were assessed by.

Immunoradiometric method according to manufacturer’s instructions.

### Sample processing

For colon tissue lysis, the full thickness colon tissue was homogenized in 1 mL cell lysis buffer containing protease inhibitor cocktails (Roche, 05056489001). The homogenate was incubated on ice for 30 min and finally the samples were centrifuged at 13,000 X g for 20 min at 4°C. Supernatants were later subjected to immunoprecipitation with the indicated antibodies in protein A/G beads.

All study samples (sera and tissue supernatant)were diluted in RIPA buffer (10 mM Tris (pH 7.4), 150 mM NaCl, 1% Triton X100, 1% Na. deoxycholate; dilution 1:1), supplemented with protease inhibitors cocktail (Sigma- Aldrich, Milano, Italy) to further proceed to Co-immunoprecipitation.

### Co-immunoprecipitation

Co-IP was performed to identify the TLR-miRNA ligand interaction using the Pierce Co-Immunoprecipitation kit (Pierce, Thermo Fisher Scientific, Illinois, USA) according to manufacturer’s instructions. Antibodies used for immunoprecipitation were TLR1 monoclonal antibody purchased from (abcam,MA,USA#ab68158), TLR7 monoclonal antibody purchased from (Cell signaling technology, Beverly, MA, USA #5632s) and TLR8 monoclonal antibody purchased from Santa-Cruz Biotechnology (Santa Cruz Biotechnology, California, USA #SC-135584). Briefly, a 50-μl aliquot of the supplied antibody-coupling gel slurry (AminoLink Plus gel) was added to the pierce spin columns and washed with the coupling buffer supplied in the kit followed by conjugation with 50 μg of primary antibodies;TLR1-ab,TLR7-ab and TLR8-ab. The primary antibodies were covalently linked to the coupling gel by the addition of sodium cyanoborohydride and left for 2 h incubation at room temperature with constant agitation. Samples were then diluted with IP Lysis/ Wash Buffer (supplied within the kit; dilution 1:1 according to manufacturer’s instructions) then added to the antibody coupled resin with a maximum of 500 μl per spin column and incubated overnight with gentle mixing at 4°C.The spin columns were then washed and eluted in 50 µl elution buffer supplied within the kit. The CO-IP elute was then divided into two halves: a half for TLR protein estimation by ELISA and the other half for miRNA ligand expression by RT-qPCR.

### ELISA measurement of TLRs protein in Co-IP eluate

The total protein in Co-IP eluate was measured using the BioRad Bradford protein assay (Biorad, CA, USA). Quantitative detection of TLRs 1,7 and 8 protein was done using (EIAab® TLR1, TLR7, TLR8 ELISA, EIAab Science Inc, Wuhan, China) kits according to manufacturer’s instructions. Luminescence signal was detected using BioTek ELX800 microplate luminescence reader (Bio-Tek Instr., Winooski, VT, USA).

### MicroRNA extraction and Protein digestion of Co-IP elute

Total RNA (including miRNAs) was isolated from Co-IP elute using reagents supplied within the miRNeasy Mini kit (Qiagen, CA, USA) in addition to proteinase K buffer (Qiagen, CA, USA) for protein digestion during RNA preparation. Briefly the was re-suspended in 150 μl proteinase K buffer and incubated at 55 °C for 30 min with shaking. After the incubation, the supernatant was transferred into 250 μl RIPA Buffer (Thermo Fisher Scientific, Illinois, USA) and 400 μl of Qiazol Lysis reagent (Qiagen, CA, USA) was added to each tube. After centrifugation, the aqueous phase was removed into a new tube, mixed with 400 μl chloroform and vortexed briefly then centrifuged to separate phases. The aqueous phase was removed into a new tube. 50 μl of RWT buffer and 30 μl of RPE buffer (Buffers of miRNeasy Mini kit, Qiagen, CA, USA) were added to enhance the precipitation of RNA at -80 °C for overnight. After centrifugation, the pellet was washed by 80% ethanol and re-suspended in 15 μl of RNase-free water as illustrated in the protocol mentioned by Wang et al., (2018) [26]. The concentration and purity of the extracted RNA were determined by Thermo Scientific NanoDrop Spectrophotometer ( Thermo Scientific, Wilmington, USA) where the RNA purity was found to be ≥ 1.8.

### Reverse Transcription-quantitative Polymerase Chain Reaction (RT-qPCR)

The extracted total RNA was reverse transcribed into cDNA using the miRCURY LNA RT kit (Qiagen, Hilden, Germany# cat no. 339340) according to manufacturer’s instructions where the thermal cycler; Thermo Hybaid PCR express (Thermo Fisher Scientific, Illinois, USA) was programmed for 60 min at 42 degrees then 5 min at 95 degrees to inactivate the transcriptase enzyme and finally cooled at 4 degrees.

The expression of 7 miRNAs was analyzed; hsa-miR-122-3p, hsa-miR-29b-3p, hsa-let-7i-3p, hsa-miR-15b-3p, hsa-miR-98-3p, hsa-miR-202-3p, hsa-miR-21-3p against endogenous control cel-mir-39 in a micro RNA array using miRCURY LNA miRNA Custom PCR Panel (Qiagen, Hilden, Germany# cat no. 339330) and miRCURY LNA SYBR Green PCR kit (Qiagen, Hilden, Germany #cat no.339345) according to manufacturer’s instructions. Due to Locked Nucleic Acid (miRCURY LNA) technology used in the primer synthesis and covered by patents owned by Qiagen, the sequences of the primers were not provided. The reaction was run on the ABI 7500 Real –Time PCR system (Applied Biosystems; Thermo Fisher Scientific, Inc., Illinois, USA). qPCR was conducted as follows: 95˚C for 2 min, 40 cycles at 95˚C for 10 s, 56˚C for 60 s and melting curve analysis at 60 ˚C -95 ˚C. Relative gene expression levels were normalized to a housekeeping gene (SNORD 68). The 2^−∆∆Ct^ equation was used to calculate the expression of the selected RNA-based candidate gene panel using the Applied Biosystems 7500 software v2.3 (Applied Biosystems; Thermo Fisher Scientific, Inc., Illinois, USA). In this study, appropriate standardization strategies were implemented according to MIQE guidelines [27].

### CRC cell lines and culture

Human CRC cell lines LS174T and HT29 were obtained from American Type Culture Collection (ATCC, Manassas, VA, USA). The cells were cultured in Dulbecco’s modified Eagle’s medium (DMEM; Invitrogen, CA, USA) as per the manufacturer’s recommendations supplemented with 10% fetal bovine serum (FBS; Invitrogen, CA, USA) and 1% streptomycin in a humidified atmosphere of 5% CO2 at 37 °C. Cells were passaged at 75% confluence with 0.02% EDTA/0.25% trypsin. Cell culture pellet would be used for Co-immunoprecipitation then TLRs protein detection by ELIZA and miRNAs by Rt-PCR as above mentioned.

Measurement of IL6 by ELISA according to the manufacturer’s instructions, the concentrations of interleukin ( IL-6), in the cell culture pellets were analyzed using an ELISA kit( Invitrogen, USA) with measurement of the optical density (OD) at 450 nm on An automated microplate reader. And the results were expressed per mg protein.

### Transfection of miRNA mimics

Three miRNA mimics of the downregulated miRNAs; miRCURY LNA miRNA mimic hsa-miR-122-3p (5'AACGCCAUUAUCACACUAAAUA3’# YM00470503), miRCURY LNA miRNA mimic hsa-miR-29b-3p (5'UAGCACCAUUUGAAAUCAGUGUU3’ # YM00473486) and miRCURY LNA miRNA mimic hsa-miR-15b-3p. (5'CGAAUCAUUAUUUGCUGCUCUA3’ # YM00472191) in addition to one negative control; miRCURY LNA miRNA mimic Negative control (UCACCGGGUGUAAAUCAGCUUG # YM00479902) were all purchased from (Qiagen, CA,USA).The tubes were centrifuged prior to opening and re-suspended by adding 300 μl RNAase-free water to the 20 nmol miRNA mimics and negative control to reach a final concentration of 20 μM.

LS174T and HT29 cells were seeded in 24-well plates at 5 × 10^4^ cells per well in triplets. After 24 h, cells were transiently transfected by using HiPerFect Transfection reagent (Qiagen, CA, USA) according to the manufacturer’s instructions. After 36 h, a second transfection was done reintroducing the same transfection complexes to the cells in order to ensure good transfection of the cells. After 72 h post the second transfection, cells were harvested and washed with PBS (Gibco; Thermo Fisher Scientific, MA, USA) and briefly centrifuged to precipitate pellet for Co-immunoprecipitation then miRNA expression analysis by quantitative RT-qPCR and supernatant for TLRs and IL6 expression by ELISA. Details of miRNA extraction, reverse transcription, RT-qPCR and ELISA methodology are as previously mentioned.

Cellular viability was determined by the MTT assay. For that purpose, both control miRNA- and targeted mimic-transfected clones were cultured in 96-well plates (5,000 cells/well) in a final volume of 100 μL to a confluence of 50%. The culture media was replaced 72 h later by the MTT reagent, which was incubated for 4 h at 37 ℃. Finally, equal volumes of dimethyl sulfoxide were added to each well to dissolve the crystals and the absorbance was measured at 570 nm, the reference wavelength being 690 nm.

### Statistical analysis

Statistical analysis was performed using the IBM SPSS Statistics for Windows, Version 25 (IBM SPSS Statistics for Windows, IBM Corporation, Armonk, NY, USA). Data were presented as the means ± standard deviation (SD) repeated in triplicates in tissue culture. A Student’s t-test was used for the statistical comparison of data. The nonparametric Kruskal–Wallis test was used for statistical comparison of the variables among the various groups where ranks were assigned. Chi-square test was also used to find out the relation between various qualitative data. Receiver‐operating characteristic (ROC) curves and the area under the ROC curve (AUC) were used to evaluate the specificity, diagnostic ability, and sensitivity of each gene to discriminate between CRC versus control. Youden index (calculated as J = Sensitivity + Specificity − 1) was used to determine the best effective diagnostic panel with the highest sensitivity and specificity [28]. Statistical differences were considered significantly if a P-value was less than 0.05 and highly significant if less than 0.01.

## Results

### Bioinformatics retrieval of CRC specific TLRs and their suspected miRNAs ligands

Applying bioinformatics analysis, three TLRs; TLR1, TLR7 and TLR8 and seven miRNAs (Hsa-miR122,hsa-miR29b,hsa-miR 15b, hsa-miR202, hsa-miR 98, hsa-miR 21 and hsa-miR let 7i) were selected from public data bases, based on high ranking score of their differential expression in CRC compared to controls. The 3 selected TLRs were found to be related to cancer and immunomodulatory pathways through their implication in positive regulation of tumor necrosis factor biosynthesis pathway, immune response activating and regulating pathway, leucocyte activation pathway involved in inflammatory response, regulation of cytokine biosynthetic pathway and pattern recognition receptor signaling pathway.

Pathway enrichment analysis of the seven miRNAs using DIANA tools database for all miRNAs [29] showed their implication in CRC and multiple cancers such as endometrial, prostate, pancreatic, Renal cell carcinoma and lung cancer. The selected miRNAs were also implicated in immunomodulatory pathways such as Transforming Growth Factor B (TGF-B) signaling pathway, FOXO signaling pathway, P53 signaling pathway, Hippo signaling pathway and AMPK signaling pathway.

Then, we searched for the possible clusters formation by the selected miRNAs in order to predict potential miRNA-CRC associations. We scanned Transcription factor and miRNA regulatory cascades in human diseases database that predicts miRNA clusters in a certain disease [30]. Our data revealed that three of the selected miRNAs; miRNA-21, miRNA-29b and miRNA-let7i could form clusters and thus exhibit high potentiality of being associated in CRC.

**Fig. 2:**
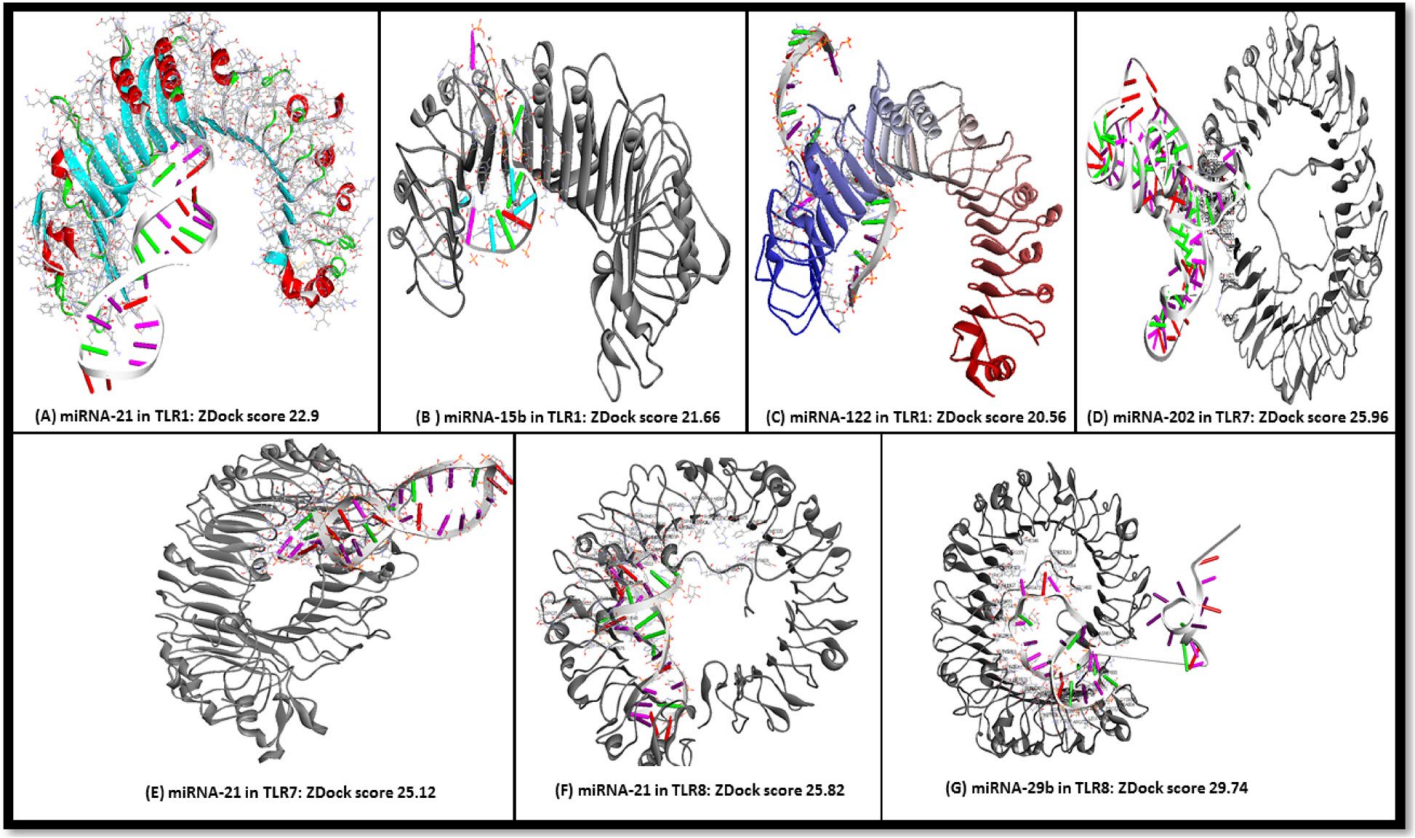
Docking of *miRNA* ligands into *TLRs* using ZDock protocol in silico (**A**) *miRNA-21* was docked into *TLR1* with ZDock score 22.9, **B** *miRNA-15b* was docked into *TLR1* with ZDock score 21.66, **C** *miRNA-122* was docked into *TLR1* with ZDock score 20.56, **D** *miRNA-202* was docked into *TLR7* with ZDock score 25.96, **E** *miRNA-21* was docked into *TLR7* with ZDock score 25.12, **F** *miRNA-21* was docked into *TLR8* with ZDock score 25.82, **G** *miRNA-29b* was docked into *TLR8* with ZDock score 29.74

Using the ZDOCK score, we performed several Docking runs; miRNA-21, miRNA-122 and miRNA-15b were docked into TLR1 then miRNA-21 and miRNA-202 were docked into TLR7 and finally miRNA-21 and miRNA-29b were docked into TLR8. The interaction analysis using ZDock and RDock revealed that miRNAs could bind to TLRs as direct ligands with high affinity in the form of high ZDock score. Specifically, TLR1 bind to miRNA-21 with highest affinity (ZDock score = 22.9, Fig. 2A), miRNA-15b (ZDock score = 21.66, Fig. 2B) and miRNA-122 with the least ZDock score (ZDock score = 20.56, Fig. 2C). On the other hand, TLR7 bind to miRNA-20 with highest affinity (ZDock score = 25.96, Fig. 2D) followed by miRNA-21 (ZDock score (ZDock score = 25.12, Fig. 2E). Notably, we found that miRNA-21 could also bind to TLR8 with higher affinity than that for TLR7 (ZDock score = 25.82, Fig. 2F). TLR8 binds with high affinity to miRNA-29b (ZDock score = 29.74, Fig. 2G). Further analysis indicated that miRNA-TLR complexes are relatively stable, and the interacting amino acids of TLRs exist at the binding interfaces with miRNAs. (More figures for Bioinformatics analysis and further details of docking interfaces are provided in Fig.S1-Fig.S24).

### Clinical, laboratory and pathological factors among the three study groups

Results of paired tissue and serum samples which were collected from patients with CRC, benign tumors and normal colonic mucosa, show significant statistical difference (*p* < 0.05) in Body Mass Index (BMI), smoking, CEA, CA19.9 the classical tumor markers of CRC, colonoscopy appearance of tumor, histological type of the tumor, Table S3.

### Differential expression of TLRs and their potential miRNA ligands in investigated groups

The protein level of TLR1, TLR7 and TLR8 in immunoprecipitated serum and tissue samples from all groups was measured using ELISA. In tissue samples, we found that TLR 1. TLR7. and TLR8 s expression is upregulated in CRC group compared to control groups (~ 6, 3, and 3 folds higher, respectively) with a highly significant statistical difference among the three groups (*p* < 0.01), Tables S4,S5.

We observed strong correlation between tissue and serum levels of all investigated TLRs and miRNAs. Altogether, these results suggest frequent communication between blood and corresponding CRC tissues and potential miRNA signature in blood, which are not random but have regular patterns. Thus, serum miRNAs could be a non-invasive biomarker for facilitating the diagnosis and prognosis of CRC.

**Fig. 3:**
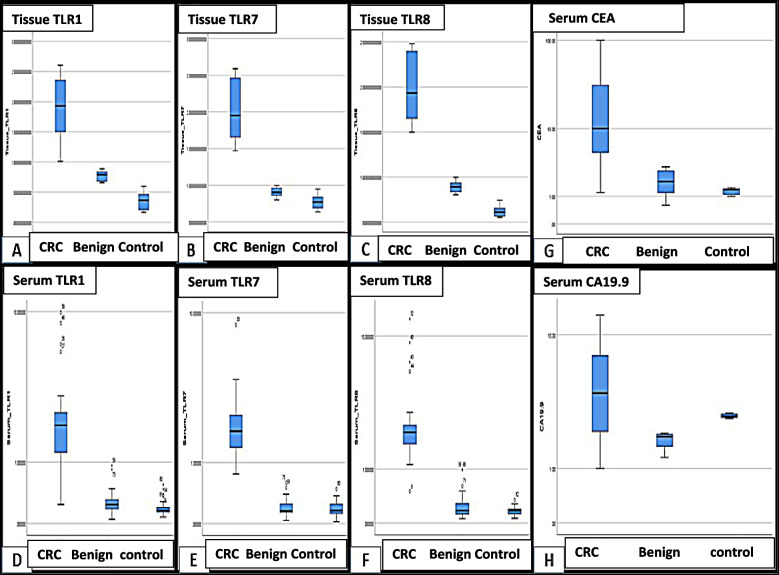
Simple BOXPLOT graph represents differential expression of TLRs in IP of tissue and serum samples of CRC versus benign and normal control cases determined by ELISA (**A**-**F**). Serum CRC tumor markers CEA (**G**) and CA19.9 in the investigated groups. The line inside the box is the median. The top and bottom lines of the box are the first and third quartiles, respectively. The top and bottom whiskers are the 5th and 95th percentiles, respectively

In 100 serum samples, expression of TLR1, TLR7, and TLR8 was found to be upregulated in CRC group compared to control groups (~ 12, 11 and 15 folds higher, respectively) (*p* < 0.01).(Tables S4 & S5, Fig. 3). ANOVA post-hoc test revealed a highly significant difference between CRC and benign groups in the three TLRs (*p* = 0.000), but a non-significant difference between healthy control and benign groups.

**Fig. 4:**
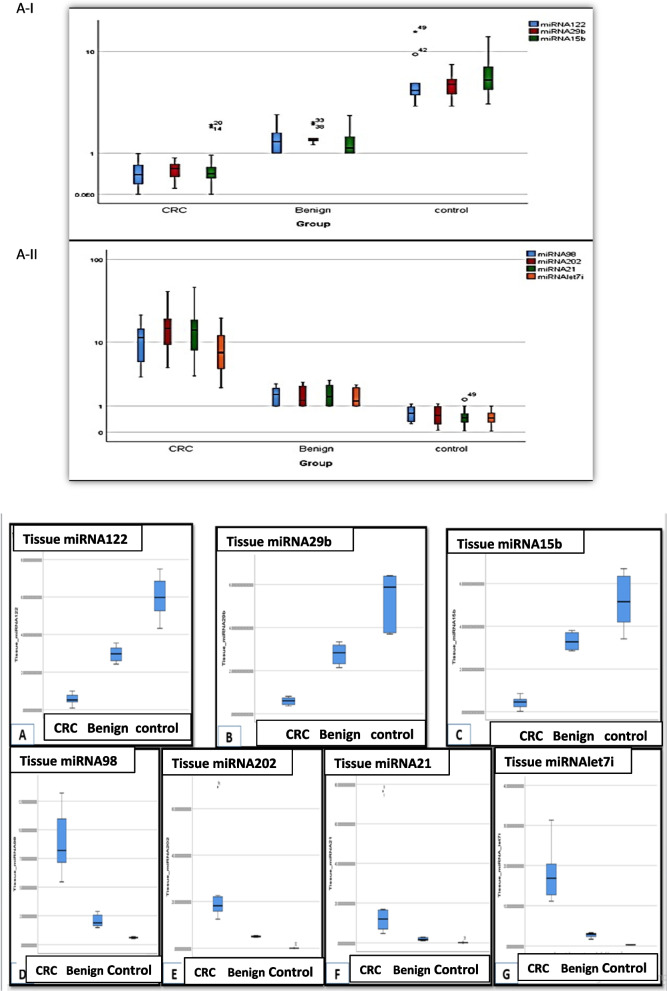
**A**-I Clustered BOXPLOT graph represents serum miRNA-122, miRNA-29b, miRNA-15b differential expression as determined by RT-qPCR among CRC, Benign adenoma and healthy control groups in IP of serum. **A**-II Clustered BOXPLOT graph represents serum miRNA-98, miRNA-202, miRNA-21, miRNA-let7i differential expression as determined by RT-qPCR among CRC, Benign adenoma and healthy control groups in IP of serum. The line inside the box is the median. The top and bottom lines of the box are the first and third quartiles, respectively. The top and bottom whiskers are the 5th and 95th percentiles, respectively. **B** Simple BOXPLOT graph represents differential expression of investigated miRNAs suggested to act as TLRs ligands in IP of tissue samples of CRC versus benign and normal control cases determined by RT-qPCR. The line inside the box is the median. The top and bottom lines of the box are the first and third quartiles, respectively. The top and bottom whiskers are the 5th and 95th percentiles, respectively

We also analyzed the miRNA expression of 7 miRNAs suspected to act as TLR ligands by RT-qPCR in the immunoprecipitate of both tissue and serum samples of the three study groups. In the initial screening set of tissue samples, three of the miRNAs (miRNA-122, miRNA-29b: and miRNA-15b) were found to be relatively downregulated in CRC group compared to Benign and normal control groups. The expression level of RQ of miRNA-122, miRNA-29b and miRNA-15b was 12, 10, 13 folds, respectively, lower in CRC group compared to control with a highly significant statistical difference among the three groups (*p* < 0.01). As for miRNAs miRNA-202, miRNA-98, miRNA 21 and miRNA-let7i, they were found to be relatively upregulated in CRC group compared to benign and normal control groups. Their fold change of expression RQ was increased by 15, 806, and 189, 88.5 times respectively in CRC group compared to control group (Tables S4 & S5, Fig. 4A).

In the clinical validation set of 100 serum samples, three of the miRNAs (miRNA-122, miRNA-29b: and miRNA-15b) were found to be relatively down regulated in CRC group compared to Benign and normal control groups where their fold change of expression RQ was decreased in CRC group 13 times compared to control with a highly significant statistical difference among the three groups (*p* < 0.01). While four miRNAs (miRNA-202, miRNA-98, miRNA 21 and miRNA-let7i) were found to be relatively upregulated in CRC group compared to benign and normal control groups. The fold change of expression RQ of miRNA-98 and miRNA-let7i was increased 21 times in CRC group compared to control group, while fold change of expression RQ was increased 29 times for miRNA-202 and miRNA-21 in CRC group compared to control group with a highly significant statistical difference among the three groups (*p* < 0.01). (Tables S4 & S5, Fig. 4B). ANOVA post-hoc test revealed a significant difference between healthy control and benign groups in miRNA-122(*p* = 0.038), and a highly significant difference between CRC and benign groups in miRNA-15b (*p* = 0.006), while a significant difference between CRC and benign groups in miRNA-202(*p* = 0.036) and miRNA-21 (*p* = 0.033).

### The discriminatory power of the investigated TLRs and their miRNA ligands

**Fig. 5:**
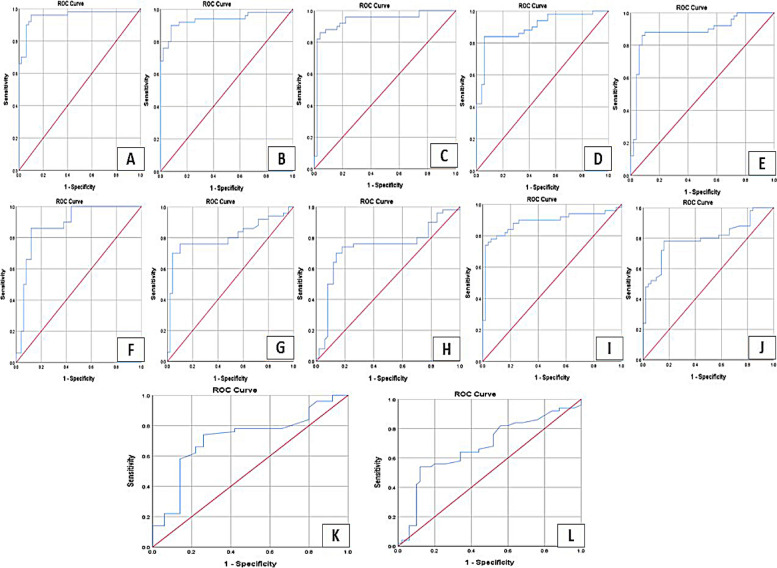
Receiver operator characteristics (ROC) curves representing the investigated serum TLRs-miRNA network discriminatory power of CRC from benign neoplasm patients and healthy volunteers. **A** *TLR1*, **B** *TLR7,* **C** *TLR8*, **D** *miRNA-122,* **E** *miRNA-29b*, **F** *miRNA-15b,* **G** *miRNA-98,* **H** miRNA-202, **I** miRNA-21, **J** miRNA-let7i, **K** CEA, and **L** CA19.9

To investigate the discriminatory power of investigated serum TLRs and their miRNA ligands in CRC patients from patients with benign colorectal adenoma and healthy volunteers, ROC curves were analyzed and AUC values were obtained (Fig. 5). The discriminatory power was found to be highest in TLR1 (AUC = 0.954, best cut off value = 0.237) followed by TLR8 (AUC = 0.937, best cut off value = 0.521), TLR7 (AUC = 0.934, best cut off value = 0.397), miRNA-122 (AUC = 0.898, best cut off value = 0.9814), miRNA-29b (AUC = 0.885, best cut off value = 1.275), miRNA-21(AUC = 0.885, best cut off value = 1.255), miRNA-15b (AUC = 0.880, best cut off value = 0.966), miRNA-98 (AUC = 0.807, best cut off value = 1.118), miRNA-let7i (AUC = 0.797, best cut off value = 1.019), miRNA-202 (AUC = 0.735, best cut off value = 1.185) and at last the classical CRC biomarkers; CEA (AUC = 0.715, best cut off value = 3.05) and CA19.9 (AUC = 0.677, best cut off value = 4.86). Hence, these optimal cutoff values of the investigated biomarkers could be used to discriminate between CRC from benign adenoma patients and healthy participants suggesting their use as powerful diagnostic biomarkers of CRC surpassing the discriminatory power of the classical biomarkers CEA and CA19.9.

### Correlation between investigated serum TLRs and their potential ligands of miRNAs

A highly significant negative correlation was found between TLR1, TLR7, TLR8 and miRNA-122, miRNA-29b and miRNA-15b (the down regulated miRNAs) while a highly significant positive correlation was found between TLR1, TLR7, TLR8 and miRNA-98, miRNA-202, miRNA-21, miRNA-let7i (the upregulated miRNAs) (*p* < 0.01) (Table S6). This implies the high possibility of miRNAs acting as direct ligands of TLRs as proposed by bioinformatics analysis described above.

### Effects of miRNA mimics of miRNA-122, miRNA-29b, miRNA-15b on CRC cell count

**Fig. 6:**
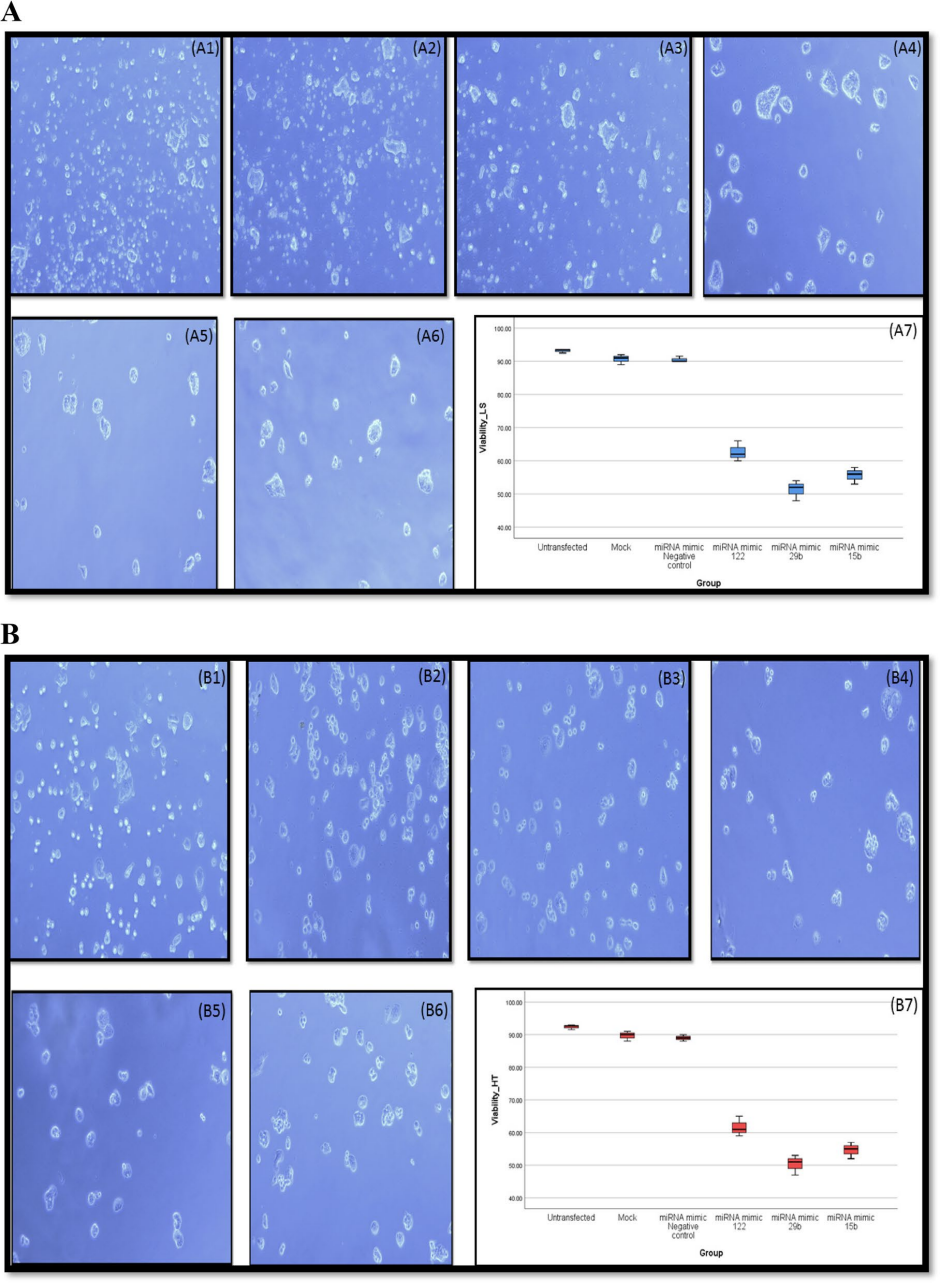
**A** LS174T CRC cell count and viability analysis 72 h post transfection showing; (A1) high cell count in the untransfected group,(A2) high cell count (minimal cytotoxicity of transfection reagent) in the mock group, (A3) high cell count in the miRNA mimic-transfected negative control group, (A4) marked reduction in cell count in the miRNA mimic-122 transfected group, (A5) marked reduction in cell count in the miRNA mimic-29b transfected group, (A6) marked reduction in cell count in the miRNA mimic-15b transfected group, (A7) Simple BOXPLOT graph represents the difference in the viability % of LS174T CRC cells among the study groups. **B** HT29 CRC cell count and viability analysis 72 h post transfection showing; (B1) high cell count in the untransfected group,(B2) high cell count (minimal cytotoxicity of transfection reagent) in the mock group, (B3) high cell count in the miRNA mimic-transfected negative control group, (B4) marked decrease in cell number in the miRNA mimic-122 transfected group, (B5) pronounced lowering in cell number in the miRNA mimic-29b transfected group, (B6) marked reduction in cell count in the miRNA mimic-15b transfected group, (B7) Simple BOXPLOT graph represents the difference in the viability % of HT29 CRC cells among the study groups

To further examine the role of differentially expressed miRNA in CRC pathogenesis, we transfected human CRC cell lines with three miRNA; miRNA-122, miRNA-29b and miRNA-15b, which were aberrantly downregulated in CRC tissue lysates compared to controls. Briefly, human CRC cell lines; LS174T and HT29 were successfully transfected with Hiperfect Transfection reagent as described under M&M and we examined the following 6 groups: untransfected (untreated) group, Mock group (only transfection reagent added to assess its cytotoxicity), group transfected with miRNA mimic negative control, group transfected with miRNA mimic of miRNA-122, group transfected with miRNA mimic of miRNA-29b and group transfected with miRNA mimic of miRNA-15b. Notably, our results indicated that higher expression of these mimics in both CRC cell lines significantly reduced cellular proliferation.

(LS174T cell line shown in Fig. 6A1-A6) and (HT-29 cell line shown in Fig. 6B1-B6) as well as viability (Fig. 6A7 in LS174T cell line; Fig. 6B7 in HT-29 cell line). The cell count was decreased in groups transfected with miRNA mimics by 2 folds when compared to mock control group (= *p* < 0.01), The percentage of viable cells was reduced by ~ twofold s in groups transfected with miRNA mimics compared to the mock group (*p* < 0.01). However, there was no significant difference as regards count between group transfected with miRNA mimic negative control and mock group (*p* = 0.108 and 0.360 respectively and between untransfected group and mock group there was no significant difference as well (*p* = 0.211 and 0.129 for count.

### Effects of miRNA mimics of miRNA-122, miRNA-29b, miRNA-15b on the viability of CRC cells

The percentage of viable cells was reduced by ~ twofold s in groups transfected with miRNA mimics compared to the mock group (*p* < 0.01). However, there was no significant difference as regards viability of cells between group transfected with miRNA mimic negative control and mock group (*p* = 0.869 and 0.567) and between untransfected group and mock group there was no significant difference as well (*p* = 0.211 and (*p* = 0.092 and 0.072).

### Effects of miRNA mimics of (miRNA-122, -29b, -15b) transfection on the expression of miRNA-TLR –IL6 in CRC cell line

Both cell lines LS174T and HT29 were successfully transfected with miRNA mimics and the expression of the investigated miRNAs that were previously downregulated in IP of serum and tissue lysates of CRC were increased by 4 times in cells transfected with miRNA mimics; miRNA-122, miRNA-29b and miRNA-15b with a highly significant difference compared to the mock group (*p* < 0.01) in LS174T and HT-29 cell lines. Their relative expression was measured in the cell pellet of harvested cells 72 h post second transfection by RT-qPCR.

**Fig. 7:**
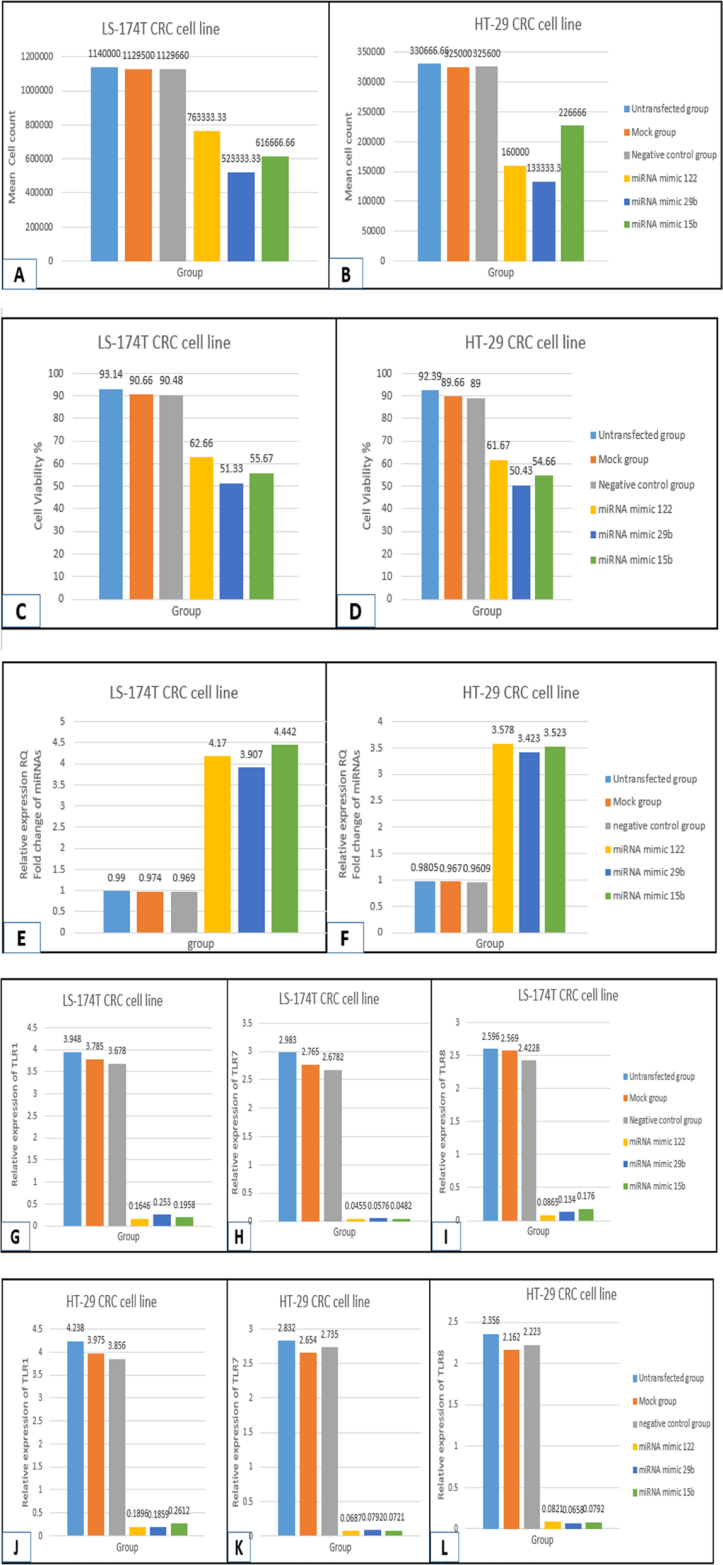
A graphical comparison among the studied groups in CRC cell line; untransfected group, mock group, negative control group, group transfected with miRNA mimic 122, group transfected with miRNA mimic 29b and group transfected with miRNA mimic 15b as regards (**A**) mean of cell count in LS-174 T CRC cell line, **B** mean of cell count in HT-29 CRC cell line, **C** cell viability percentage in LS-174 T cell line, **D** cell viability percentage in HT-29 cell line, **E** Fold change RQ of the three investigated miRNAs; miR-122, miR-29b, miR-15b by RT-qPCR in LS-174 T cell line, **F **Fold change RQ of the three investigated miRNAs; miR-122, miR-29b, miR-15b by RT-qPCR in HT-29 cell line, **G** Relative protein expression of TLR1 by ELISA in LS-174 T cell line, **H** Relative protein expression of TLR7 by ELISA in LS-174 T cell line, **I **Relative protein expression of TLR8 by ELISA in LS-174 T cell line, **J **Relative protein expression of TLR1 by ELISA in HT-29 cell line, **K** Relative protein expression of TLR7 by ELISA in HT-29 cell line, **L** Relative protein expression of TLR8 by ELISA in HT-29 cell line

As for the TLRs; TLR1, TLR7 and TLR8 that were previously significantly upregulated in IP of serum and tissue lysates of CRC were found to be significantly decreased by 19 times for TLR1 as well as TLR8 and 57 times for TLR7 in cells transfected with miRNA mimics; miRNA-122, miRNA-29b and miRNA-15b with a highly significant difference compared to the mock group (*p* < 0.01) in LS174T and HT-29 cell lines. Their relative expression was measured in the supernatant of harvested cells 72 h post second transfection by ELISA (Fig. 7). On the other hand, IL6 protein expression -the target effector of this signal pathway- was significantly lowered in both cell lines after miRNA transfection (Table 1).

**Table 1:** IL6 protein expression in both cell lines G&H before and after miRNA transfection

Group	(G) IL6 protein expression (pg/mg protein) by ELISA in LS174T CRC cell line			(H) IL6 protein expression (pg/mg protein) by ELISA in HT-29 CRC cell line		
	Mean	SD	*p*	Mean	SD	*p*
**Untransfected group**	525	48. 99	0.094^**(a)**^	324. 83	23. 026	0.072^**(a)**^
**Mock group**	480	67. 16	––-	309. 71	18. 23	–––
**Negative Control**	460	63. 21	0.437^**(b)**^	288. 97	28. 33	0.688^**(b)**^
**miRNA mimic -122**	230	34. 74	0.000^(c)**^	150. 09	16. 27	0.000^(c)**^
**miRNA mimic- 29b**	188	23.04	0.001^(d)**^	134.01259	20. 09	0.006^(d)**^
**miRNA mimic -15b**	230	30.06	0.000^(e)**^	160. 25	11.9	0.001^(e)**^

^a^Independent samples t-test (untransfected group versus mock group)

^b^Independent samples t-test (Negative control group versus mock group)

^c^Independent samples t-test (miRNA mimic-122 transfected group versus mock group)

^d^Independent samples t-test (miRNA mimic-29b transfected group versus mock group)

^e^Independent samples t-test (miRNA mimic-15b transfected group versus mock group)

p: *NS* Not significant (> 0.05), **p < 0.01: highly significant

## Discussion

Increasing evidence has suggested the new role of miRNAs as direct ligands of TLRs besides their classical role of regulation of gene expression [11]. However, the effects of miRNA-TLRs signaling on CRC pathogenesis are still poorly understood. In the present study, our in silico analysis identified, for the first time, three TLRs (TLR1, TLR7 and TLR8) and seven miRNAs (hsa-miR-122-3p, hsa-miR-29b-3p, hsa-let-7i-3p, hsa-miR-15b-3p, hsa-miR-98-3p, hsa-miR-202-3p, hsa-miR-21-3p) as differentially expressed molecules in CRC compared to controls. We have also assessed the possibility of binding between the selected TLRs and miRNAs as direct ligands through insilico molecular docking ZDock protocol.

Recently, computational strategies helped greatly in novel drug design of which molecular docking; a well-established computational method of drug discovery; is widely used to predict how a ligand interacts with its receptor and how tightly it binds revealing the electrostatic and steric complementarity between the protein and the ligand [31]. Also, the Prediction of miRNA ligand interaction with receptor protein is highly desirable to design miRNA-based therapeutics [32]*.* In this study, we introduced ZDock protocol approach of molecular docking of miRNAs into TLRs protein predicting the direct interaction between them. After performing several runs for docking the selected miRNAs into the selected TLRs; we found that relatively stable complexes could be formed by miRNA ligand binding to TLR with relatively high affinity as per high ZDock score.

Our in insilico analysis was consistent with in vivo expression of these TLRs and miRNA in serum and tissue samples from CRC patients.

We found the protein expression of TLR1, TLR7 and TLR8 to be aberrantly upregulated in CRC cases compared to their protein expression in benign neoplasms cases and healthy control samples in agreement to the studies of [33] who reported the up regulation of TLR1, TLR7 and TLR8 in CRC patients in comparison to control individuals [14]. On the other hand, we measured the expression of 7 miRNAs proposed or hypothesized to act as TLRs ligands by RT-qPCR and found that three of them (miRNA-122, miRNA-29b and miRNA-15b) were downregulated in CRC cases compared to benign cases and healthy normal control, while the other four (miRNA-202, miRNA-98, miRNA 21, miRNA-let7i) were found to be upregulated.

Several studies support our findings [15–17]*.* Although previous reports confirmed the differential expression of miRNA 122 in colorectal cancer versus normal control which was proved in our study, Sun et al., [34] found that exosomal miRNA-122 was significantly overexpressed in CRC patients especially in those with liver metastasis [34]. Also, Maierthaler et al., [35] defined a clear association of miR‐122 levels to the occurrence of liver metastasis in CRC patients [35]. Moreover, Pan et al., [36] demonstrated the upregulation of miRNA-15b in CRC cases in comparison to normal control [36]. This discrepancy is mostly attributed to the presence of liver metastasis associated with CRC patients in these previous reports since miR-122 is a liver specific miRNA that is highly enriched within hepatocytes. Also, liver metastasis with other types of cancer e.g. gastric cancer is associated with overexpression of miRNA 122 which has been a reported as a useful marker to differentiate colorectal with liver metastasis versus those without [34, 37]. It could also be also attributed to population differences that were observed for complex traits which may be due to the combined effect of socioeconomic, environmental, genetic and epigenetic factors [38]. Moreover, variations in biological samples type (exosome, serum, tissue), samples processing and RNA quantitation methods are responsible for discrepancy in miRNAs expression between different studies [39].

Accumulating evidence reported the upregulated expression of (miRNA-202, miRNA-98, miRNA 21, miRNA-let7i) in CRC cases compared to benign cases and healthy normal control [18–21].In contrast, Lin et al. [40] Zhu et al. [41], and Song et al. [42], reported the downregulated expression of miR-202, miR-98 and let7i in CRC tissues compared to adjacent colon tissues respectively [40–42]*.*

In the light of previous findings We tend to focus on miRNAs role in CRC beyond the post-transcriptional level by serving as ligands of TLRs [8, 43]. Based on our study docking results, that verified the stability of miRNA-TLR complexes, we postulated that miRNA-122, -29b, and-15b can act as a signaling antagonist of TLR 1and -8 because the interacting amino acids of TLRs exist at the binding interfaces with miRNAs sequence in agreement with Heil approach [44]. These insilico results were validated by coimmunoprecipitation of TLRs-miRNA complexes from serum and tissue samples. Thus, synthetic miRNA-122, -29b, and-15b may act both as paracrine antagonists and transcription suppressors of TLRs 1,7 and 8 receptor protein inhibiting macrophages via suppressing MyD88 thus inhibiting the formation of a complex with TRAF6, IRAK1, IRAK4 that leads to NF- kappa B suppression [45], and inhibiting proinflammatory cytokines secretion (e.g., TNF-α and IL-6). The major sources of IL-6 in CRC are tumor-associated mesenchymal stem cells, macrophages and colon cancer-associated stromal fibroblasts. TLR1,7,8 inhibition ultimately leads to cessation of Epithelial to Leucocytic transition. Epithelial to leucocytic transition (ELT) is the acquisition of immune properties by tumor cells. Epithelial tumor cells can make transition to a mesenchymal phenotype which increases local motility and remodeling of the extracellular matrix This will leads to tumor growth inhibition that go in hand with Luddy and his colleagues [46].

## Conclusion

**Fig. 8:**
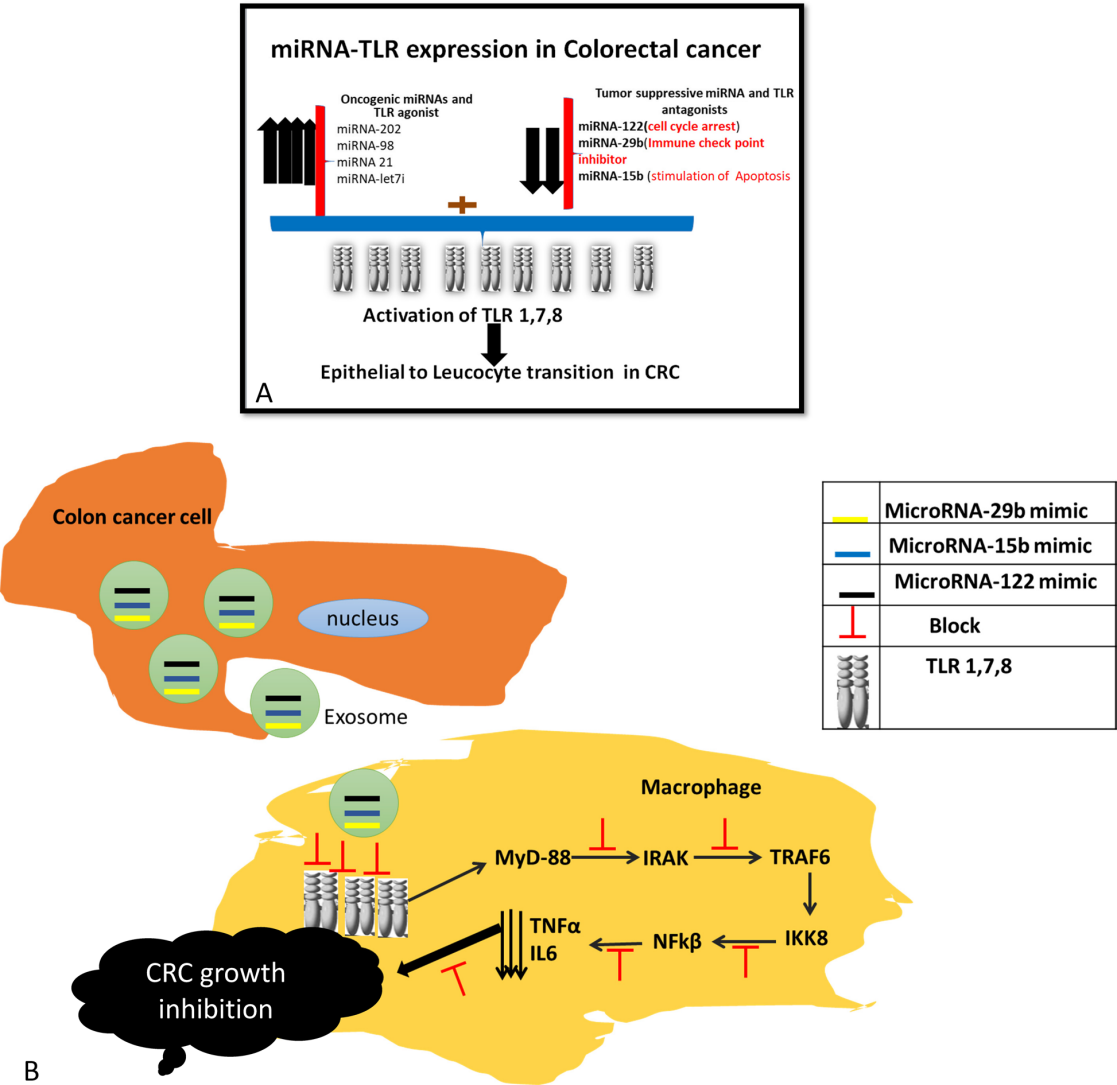
Proof of concept of study hypothesis. **A** CRC patients showed upregulation of oncogenic miRNA-202,-98, 21, and let7i and downregulation of tumor suppressive miRNA-122, -29b, and-15b with subsequent activation of TLR1,7, 8 that resulted in CRC pathogenesis by epithelial to leukocytes transition is the acquisition of immune properties by tumor cells (1) evade the immune system at the primary tumor site, (2) access the lymphatic system, (3) circulate through the vasculature, home to favorable sites of metastasis, and extravasate into a metastatic niche, and (4) avoid destruction by the immune system at the site of metastasis. **B** Proof of concept of study hypothesis: miRNA-122, -29b, and-15b act as antagonists of TLR 1,7 ,8 with suppressing MyD88 and inhibiting the formation of a complex with TRAF6, IRAK1, IRAK4 that leads to NF- kappa B and TNFα suppression with CRC tumor growth inhibition

The present study proposed a novel approach that enables a reliable integration of Toll like receptors and miRNAs in CRC management. We hypothesized that miRNAs act as TLR ligands where they block or inhibit pro-tumorigenic TLR1/7/8-mediated tumor growth and survival in CRC (Fig. 8).

### Supplementary Information

Supplementary file: Table S1 and Figure S1: Figure S23.

## Data Availability

Data is provided within the manuscript or supplementary information files.
