# Supplementary material for: miRNAs: possible regulators of toll like receptors and inflammatory tumor microenvironment in colorectal cancer
Source: BMC Cancer. 2024 Jul 10;24:824. doi: 10.1186/s12885-024-12417-0 (PMC11238347; doi:10.1186/s12885-024-12417-0)
Supplement: Supplementary file 1 — Supplementary file: Table S1 and Figure S1: Figure S23. [file 12885_2024_12417_MOESM1_ESM.docx]

##

**Supplementary file:**

- **Supplementary table S1 :TLR Expression in 2478 large intestine with percent of mutation and copy number variation**

| **Cancer type** | **TLR1** | **TLR7** | **TLR8** |
| --- | --- | --- | --- |
| colorectal adenocarcinoma  total number of tested samples (1519) samples | **17** | **28** | **34** |

- **Supplementary table S2 :Number of samples of colorectal adenocarcinoma with a mutation in TLR 1,7 & 8 genes in the COSMIC da**tabase.

| **TLR** | **Point Mutations** | | **Copy Number Variation** | | **Gene Expression** | | **Gene Expression** | |
| --- | --- | --- | --- | --- | --- | --- | --- | --- |
|  | **Percent mutant** | **Tested** | **Percent variant** | **Tested** | **Percent regulated** | **Tested** | **Percent methylated** | **Tested** |
| **TLR1** | **49** | **2478** | **1** | **718** | **19** | **610** | **-** | **-** |
| TLR7 | 71 |  | 6 |  | 18 |  | - | - |
| TLR8 | 90 |  |  |  | 6 |  | - | - |

- Entries larger than 5 are highlighted in bold."

**Supplementary Table S3:** Clinical - pathological factors and laboratory parameters in different groups of the study:

| **Clinical-pathological Factors in initial screening set**  **(Total 32 paired serum and tissue samples)** | | | | | |
| --- | --- | --- | --- | --- | --- |
|  | **Colorectal cancer** | **Benign colorectal neoplasm** | **Healthy Control** | **P** | **χ^2^**  **^(a)^** |
|  | **(N=16) (%)** | **(N=8) (%)** | **(N=8) (%)** |  |  |
| **Age:**  **≥** 46.2 years (15)  **<**46.2 years (17) | 10 (62.5%)  6 (37.5%) | 3 (37.5%)  5 (62.5%) | 2 (25%)  6 (75%) | 0.184  NS | 3.388 |
| **Sex:**  Male (20)  Female (12) | 10 (62.5%)  6 (37.5%) | 6 (75%)  2 (25%) | 4 (50%)  4 (50%) | 0.587  NS | 1.067 |
| **Smoking:**  Smoker (11)  Non-Smoker (21) | 10 (62.5%)  6 (37.5%) | 1 (12.5%)  7 (87.5%) | 0 (0%)  8 (100%) | 0.003**  **HS** | 11.498 |
| **BMI:**  Underweight (1)  Normal (23)  Overweight (8) | 0 (0%)  8 (50%)  8 (50%) | 1 (12.5%)  7 (87.5%)  0 (0%) | 0 (0%)  8 (100%)  0 (0%) | 0.010*  **S** | 13.217 |
| **Family History**  Positive (4)  Negative (28) | 3 (18.8%)  13 (81.3%) | 1 (12.5%)  7 (87.5%) | 0 (0%)  8 (100%) | 0.424  NS | 1.714 |
| **Colonoscopy**  Negative (25)  Mass (10)  Polyp (12)  Other (2) | 0 (0%)  10 (62.5%)  4 (25%)  2 (12.5%) | 0 (0%)  0 (0%)  8 (100%)  0 (0%) | Safety margins  8 (100%)  0 (0%)  0 (0%)  0 (0%) | 0.002**  **HS** | 12.< 0.01 |
| **Histological Type**  Adenocarcinoma  Hyperplastic Polyp  Tubular | 16 (100%)  0 (00%)  0(0%) | 0 (0%)  4 (50%)  4 (50%) | 0 (0%)  0 (0%)  0 (0%) | < 0.01**  **HS** | 24.< 0.01 |
| **Laboratory Parameters H^(b)^** | | | | | |
| **CEA (ng/ml)**  Median  Mean Rank | 10.150  23.06 | 1.905  12.00 | 1.360  7.88 | < 0.01**  **HS** | 16.473 |
| **CA19.9 (u/ml)**  Median  Mean Rank | 4.450  19.50 | 2.< 0.01  8.00 | 2.895  19.00 | 0.012^*^  **S** | 8.799 |
| **Clinical-pathological Factors in clinical validation set**  **(Total 100 serum samples)** | | | | | |
|  | **Colorectal cancer** | **Benign colorectal neoplasm** | **Healthy Control** | **P** | **χ^2^**  **^(a)^** |
|  | **(N=50) (%)** | **(N=25) (%)** | **(N=25) (%)** |  |  |
| **Age:**  **≥** 46.2 years (60)  **<**46.2 years (40) | 31 (62%)  19 (38%) | 17 (68%)  8 (32%) | 12 (48%)  13 (52%) | 0.325  NS | 2.250 |
| **Sex:**  Male (56)  Female (44) | 27 (54%)  23 (46%) | 12 (48%)  13 (52%) | 17 (68%)  8 (32%) | 0.334  NS | 2.192 |
| **Smoking:**  Smoker (26)  Non-Smoker (74) | 16 (32%)  34 (68%) | 4 (16%)  21 (84%) | 6 (24%)  19 (76%) | 0.319  NS | 2.287 |
| **BMI:**  Underweight (7)  Normal (78)  Overweight (15) | 7 (14%)  30 (60%)  13(26%) | 0 (0%)  23 (92%)  2 (8%) | 0 (0%)  25 (100%)  0 (0%) | 0.001*  **HS** | 19.856 |
| **Family History**  Positive (23)  Negative (77) | 9 (18%)  41 (82%) | 6 (24%)  19 (76%) | 8 (32%)  17 (68%) | 0.394  NS | 1.863 |
| **Colonoscopy**  Negative (25)  Mass (30)  Polyp (32)  Other (13) | 0 (0%)  26 (52%)  13 (26%)  11 (22%) | 0 (0%)  4 (16%)  19 (76%)  2 (8%) | 25 (100%)  0 (0%)  0 (0%)  0 (0%) | < 0.01*  **HS** | 122.734 |
| **Histological Type**  Adenocarcinoma  Mucinous adenocarcinoma  Signet ring  Hyperplastic Polyp  Tubular  Villous | 37 (74%)  10 (20%)  2 (4%)  1(2%)  0  0 | 0  0  0  8 (32%)  13 (52%)  4 (16%) | 0  0  0  0  0  0 | < 0.01**  **HS** | 71.00 |
| **Laboratory Parameters H^(b)^** | | | | | |
| **CEA (ng/ml)**  Median  Mean Rank | 4.2  61.27 | 2.4  47.48 | 1.6  31.98 | < 0.01**  **HS** | 17.394 |
| **CA19.9 (u/ml)**  Median  Mean Rank | 5.15  61.04 | 4.42  46.92 | 2.8  33.00 | < 0.01^*^  **HS** | 16.095 |

a Chi- square test (χ2)

b Kruskal-Wallis test (H)

p: NS: non- significant (>0.05), *p<0.05: significant (S), **p < 0.01:highly significant (HS).

BMI: Body mass index, CEA: Carcinoemberyonic antigen, CA19.9: Carbohydrate antigen.

**Table S4:** Differential expression of investigated biomarkers among the three study groups **in tissue samples (n=32 )**

|  | | **Mean Rank** | **H** | **F** | ***p*** |
| --- | --- | --- | --- | --- | --- |
| TLR1  Pg/mg protein | CRC (n=16) | 24.50 | 26.182 | 46.071 | 0.000 |
|  | Benign adenoma (n=8) | 12.50 |  |  |  |
|  | Normal mucosa (n=8)_ | 4.50 |  |  |  |
| TLR7  Pg/mg protein | CRC(n=16) | 24.50 | 24.909 | 60.351 | 0.000 |
|  | Benign adenoma(n=8) | 11.50 |  |  |  |
|  | Normal mucosa (n=8) | 5.50 |  |  |  |
| TLR8  Pg/mg protein | CRC(n=16) | 24.50 | 26.191 | 88.339 | 0.000 |
|  | Benign adenoma(n=8) | 12.50 |  |  |  |
|  | Normal mucosa (n=8) | 4.50 |  |  |  |
| miRNA-122-5p  (RQ=fold change) | CRC(n=16) | 0.4 | 26.220 | 214.189 | 0.000 |
|  | Benign adenoma(n=8) | 0.7 |  |  |  |
|  | Normal mucosa (n=8) | 1 |  |  |  |
| miRNA-29b-5p  (RQ=fold change) | CRC(n=16) | 0.4 | 26.230 | 127.015 | 0.000 |
|  | Benign adenoma(n=8) | 0.7 |  |  |  |
|  | Normal mucosa (n=8) | 1 |  |  |  |
| miRNA-15b-5p  (RQ=fold change) | CRC(n=16) | 0.3 | 24.946 | 141.291 | 0.000 |
|  | Benign adenoma(n=8) | 0.8 |  |  |  |
|  | Normal mucosa (n-8) | 1.1 |  |  |  |
| miRNA-98-5p  (RQ=fold change) | CRC(n=16) | 5.5 | 26.220 | 67.119 | 0.000 |
|  | Benign adenoma(n=8) | 2.7 |  |  |  |
|  | healthy control(n=8) | 0.9 |  |  |  |
| miRNA-202-5p  (RQ=fold change) | CRC(n=16) | 5.5 | 26.220 | 11.719 | 0.000 |
|  | Benign adenoma(n=8) | 2.7 |  |  |  |
|  | healthy control(n=8) | 0.9 |  |  |  |
| miRNA-21-5p  (RQ=fold change) | CRC(n=16) | 5.5 | 26.220 | 4.788 | 0.016 |
|  | Benign adenoma(n=8) | 2.7 |  |  |  |
|  | healthy control(n=8) | 0.9 |  |  |  |
| miRNA-let7i-5p  (RQ=fold change) | CRC (n=16) | 5.5 | 26.220 | 50.958 | 0.000 |
|  | Benign adenoma(n=8) | 2.7 |  |  |  |
|  | healthy control(n=8) | 0.9 |  |  |  |

*p*-value > 0.05 is considered statistically non-significant, *p*-value < 0.05 is considered significant statistically significant, and *p*-value < 0.01 is considered highly statistically significant, H: Kruskal Wallis test , F: One way ANOVA test, CEA: Carcinoemberyonic antigen, CA19.9: Carbohydrate antigen.

**Table S5:** Differential expression of investigated biomarkers among the three study groups **in sera of investigated groups (N=100)**

|  | | **Mean Rank** | **H** | **F** | ***p*** |
| --- | --- | --- | --- | --- | --- |
| TLR1  pg/ml | CRC (N=50) | 75.10 | 73.476 | 33.284 | < 0.01 |
|  | Benign adenoma(N=25) | 31.04 |  |  |  |
|  | healthy control (N=25) | 20.76 |  |  |  |
| TLR7  pg/ml | CRC(N=50) | 75.50 | 74.313 | 59.694 | < 0.01 |
|  | Benign adenoma | 26.44 |  |  |  |
|  | healthy control(N=25) | 24.56 |  |  |  |
| TLR8  pg/ml | CRC(N=50) | 75.40 | 73.701 | 34.907 | < 0.01 |
|  | Benign adenoma(N=25) | 26.20 |  |  |  |
|  | healthy control(N=25) | 25.00 |  |  |  |
| CEA  ng/ml | CRC(N=50) | 61.27 | 17.394 | 3.981 | 0.022 |
|  | Benign adenoma(N=25) | 47.48 |  |  |  |
|  | healthy control(N=25) | 31.98 |  |  |  |
| CA19.9  ng/ml | CRC(N=50) | 59.34 | 13.674 | 3.299 | 0.041 |
|  | Benign adenoma(N=25) | 50.24 |  |  |  |
|  | healthy control(N=25) | 33.08 |  |  |  |
| miRNA-122-5p  (RQ=fold change) | CRC(N=50) | 0.35 | 58.776 | 3.312 | < 0.01 |
|  | Benign adenoma(N=25) | 0.66 |  |  |  |
|  | healthy control(N=25) | 1 |  |  |  |
| miRNA-29b-5p  (RQ=fold change) | CRC (N=50) | 0.39 | 48.434 | 3.437 | < 0.01 |
|  | Benign adenoma(N=25) | 0.78 |  |  |  |
|  | healthy control(N=25) | 1.15 |  |  |  |
| miRNA-15b-5p  (RQ=fold change) | CRC(N=50) | 0.37 | 54.622 | 5.732 | < 0.01 |
|  | Benign adenoma(N=25) | 0.66 |  |  |  |
|  | healthy control(N=25) | 1.19 |  |  |  |
| miRNA-98-5p  (RQ=fold change) | CRC(N=50) | 2.1 | 23.949 | 0.695 | < 0.01 |
|  | Benign adenoma(N=25) | 1.4 |  |  |  |
|  | healthy control(N=25) | 1 |  |  |  |
| miRNA-202-5p  (RQ=fold change) | CRC(N=50) | 1.7 | 13.532 | 2.258 | 0.001 |
|  | Benign adenoma(N=25) | 1.19 |  |  |  |
|  | healthy control(N=25) | 0.98 |  |  |  |
| miRNA-21-5p  (RQ=fold change) | CRC(N=50) | 3.04 | 47.508 | 3.792 | < 0.01 |
|  | Benign adenoma(N=25) | 1.65 |  |  |  |
|  | healthy control(N=25) | 1 |  |  |  |
| miRNA-let7i-5p  (RQ=fold change) | CRC(N=50) | 3.09 | 37.512 | 0.736 | < 0.01 |
|  | Benign adenoma(N=25) | 2.3 |  |  |  |
|  | healthy control(N=25) | 1.13 |  |  |  |

*p*-value > 0.05 is considered statistically non-significant, *p*-value < 0.05 is considered significant statistically significant, and *p*-value < 0.01 is considered highly statistically significant, H: Kruskal Wallis test , F: One way ANOVA test, CEA: Carcinoemberyonic antigen, CA19.9: Carbohydrate antigen.

**Table S6.** Spearman Correlation between the investigated TLRs and miRNAs in the colon tissue and serum samples in the malignant group.

| A-Spearman Correlation between the investigated TLRs and miRNAs in the colon tissue versus serum samples in the malignant group. | | | | | | | | | | | | | | |
| --- | --- | --- | --- | --- | --- | --- | --- | --- | --- | --- | --- | --- | --- | --- |
|  | | | | Tissue R122 | Tissue miR29b | | Tissue miR15b | Tissue miR98 | TISSUS miR202 | tissue miR21 | tissue  miRlet7i | tissue TLR1 | tissue TLR7 | tissue_TLR8 |
|  | Serum miR122 | Corre.Coefficient | | **0.98**** | .594^**^ | | .687^**^ | -.384^**^ | -.224^*^ | -.490^**^ | -.616^**^ | -.615^**^ | -.505^**^ | -.521^**^ |
|  |  | Sig. (2-tailed) | | 0.00. | .< 0.01 | | .< 0.01 | .< 0.01 | .025 | .< 0.01 | .< 0.01 | .< 0.01 | .< 0.01 | .< 0.01 |
|  | Serum miR29b | Corre.Coefficient | | .594^**^ | **0.97**** | | .504^**^ | -.352^**^ | -.397^**^ | -.408^**^ | -.459^**^ | -.503^**^ | -.457^**^ | -.458^**^ |
|  |  | Sig. (2-tailed) | | .< 0.01 | < 0.01. | | .< 0.01 | .< 0.01 | .< 0.01 | .< 0.01 | .< 0.01 | .< 0.01 | .< 0.01 | .< 0.01 |
|  | Serum miR15b | Corre.Coefficient | | .687^**^ | .504^**^ | | **0.99**** | -.212^*^ | -.233^*^ | -.413^**^ | -.455^**^ | -.666^**^ | -.527^**^ | -.507^**^ |
|  |  | Sig. (2-tailed) | | .< 0.01 | .< 0.01 | | 0.00. | .034 | .019 | .< 0.01 | .< 0.01 | .< 0.01 | .< 0.01 | .< 0.01 |
|  | Serum miR98 | Corre.Coefficient | | -.384^**^ | -.352^**^ | | -.212^*^ | **0.99** | .491^**^ | .588^**^ | .328^**^ | .428^**^ | .400^**^ | .416^**^ |
|  |  | Sig. (2-tailed) | | .< 0.01 | .< 0.01 | | .034 | 0.00. | .< 0.01 | .< 0.01 | .001 | .< 0.01 | .< 0.01 | .< 0.01 |
|  |  |  |  |  |  |  |  |  |  |  |  |  |  |  |
|  | Serum miR202 | Corre.Coefficiet | -.224^*^ | | | -397^**^ | -.233^*^ | .491^**^ | **0.89** | .561^**^ | .389^**^ | .359^**^ | .309^**^ | .464^**^ |
|  |  | Sig. (2-tailed) | .025 | | | .< 0.01 | .019 | .< 0.01 | < 0.01. | .< 0.01 | .< 0.01 | .< 0.01 | .002 | .< 0.01 |
|  | Serum miR21 | Corre.Coefficiet | -.490^**^ | | | -408^**^ | -.413^**^ | .588^**^ | .561^**^ | **0.96**** | .507^**^ | .534^**^ | .497^**^ | .501^**^ |
|  |  | Sig. (2-tailed) | .< 0.01 | | | .< 0.01 | .< 0.01 | .< 0.01 | .< 0.01 | 0.00. | .< 0.01 | .< 0.01 | .< 0.01 | .< 0.01 |
|  | Serum miRLet7i | Corre.Coefficiet | -.616^**^ | | | -459^**^ | -.455^**^ | .328^**^ | .389^**^ | .507^**^ | **0.98**** | .505^**^ | .379^**^ | .413^**^ |
|  |  | Sig. (2-tailed) | .< 0.01 | | | .< 0.01 | .< 0.01 | .001 | .< 0.01 | .< 0.01 | 0.00. | .< 0.01 | .< 0.01 | .< 0.01 |
|  | Serum TLR1 | Corre.Coefficiet | -.615^**^ | | | -503^**^ | -.666^**^ | .428^**^ | .359^**^ | .534^**^ | .505^**^ | **0.99**** | .655^**^ | .626^**^ |
|  |  | Sig. (2-tailed) | .< 0.01 | | | .< 0.01 | .< 0.01 | .< 0.01 | .< 0.01 | .< 0.01 | .< 0.01 | 0.00. | .< 0.01 | .< 0.01 |
|  | Serum TLR7 | Corre.Coefficiet | -.505^**^ | | | -457^**^ | -.527^**^ | .400^**^ | .309^**^ | .497^**^ | .379^**^ | .655^**^ | **0.96**** | .559^**^ |
|  |  | Sig. (2-tailed) | .< 0.01 | | | .< 0.01 | .< 0.01 | .< 0.01 | .002 | .< 0.01 | .< 0.01 | .< 0.01 | 0.00. | .< 0.01 |
|  | Serum TLR8 | Corre.Coefficiet | -.521^**^ | | | -458^**^ | -.507^**^ | .416^**^ | .464^**^ | .501^**^ | .413^**^ | .626^**^ | .559^**^ | **0.98**** |
|  |  | Sig. (2-tailed) | .< 0.01 | | | .< 0.01 | .< 0.01 | .< 0.01 | .< 0.01 | .< 0.01 | .< 0.01 | .< 0.01 | .< 0.01 | 0.00. |
| **. Correlation is significant at the 0.01 level (2-tailed). | | | | | | | | | | | | | | |
| *. Correlation is significant at the 0.05 level (2-tailed). | | | | | | | | | | | | | | |

| B-Spearman Correlation between the investigated TLRs - miRNAs network in the serum of CRC. | | | | | | | | | | | | |
| --- | --- | --- | --- | --- | --- | --- | --- | --- | --- | --- | --- | --- |
|  | | | miR122 | miR29b | miR15b | miR98 | miR202 | miR21 | miRlet7i | TLR1 | TLR7 | TLR8 |
| **Serum Parameters** | miR122 | Cor.Coeff. | 0.98** | .594^**^ | .687^**^ | -.384^**^ | -.224^*^ | -.490^**^ | -.616^**^ | -.615^**^ | -.505^**^ | -.521^**^ |
|  |  | Sig. (2-tailed) | 0.00. | .< 0.01 | .< 0.01 | .< 0.01 | .025 | .< 0.01 | .< 0.01 | .< 0.01 | .< 0.01 | .< 0.01 |
|  | miR29b | Cor.Coeff. | .594^**^ | 0.97** | .504^**^ | -.352^**^ | -.397^**^ | -.408^**^ | -.459^**^ | -.503^**^ | -.457^**^ | -.458^**^ |
|  |  | Sig. (2-tailed) | .< 0.01 | < 0.01. | .< 0.01 | .< 0.01 | .< 0.01 | .< 0.01 | .< 0.01 | .< 0.01 | .< 0.01 | .< 0.01 |
|  | miR15b | Cor.Coeff. | .687^**^ | .504^**^ | 0.99** | -.212^*^ | -.233^*^ | -.413^**^ | -.455^**^ | -.666^**^ | -.527^**^ | -.507^**^ |
|  |  | Sig. (2-tailed) | .< 0.01 | .< 0.01 | 0.00. | .034 | .019 | .< 0.01 | .< 0.01 | .< 0.01 | .< 0.01 | .< 0.01 |
|  | miR98 | Cor.Coeff. | -.384^**^ | -.352^**^ | -.212^*^ | 0.99 | .491^**^ | .588^**^ | .328^**^ | .428^**^ | .400^**^ | .416^**^ |
|  |  | Sig. (2-tailed) | .< 0.01 | .< 0.01 | .034 | 0.00. | .< 0.01 | .< 0.01 | .001 | .< 0.01 | .< 0.01 | .< 0.01 |
|  | miR202 | Cor.Coeff. | -.224^*^ | -.397^**^ | -.233^*^ | .491^**^ | 0.89 | .561^**^ | .389^**^ | .359^**^ | .309^**^ | .464^**^ |
|  |  | Sig. (2-tailed) | .025 | .< 0.01 | .019 | .< 0.01 | < 0.01. | .< 0.01 | .< 0.01 | .< 0.01 | .002 | .< 0.01 |
|  | miR21 | Cor.Coeff. | -.490^**^ | -.408^**^ | -.413^**^ | .588^**^ | .561^**^ | 0.96** | .507^**^ | .534^**^ | .497^**^ | .501^**^ |
|  |  | Sig. (2-tailed) | .< 0.01 | .< 0.01 | .< 0.01 | .< 0.01 | .< 0.01 | 0.00. | .< 0.01 | .< 0.01 | .< 0.01 | .< 0.01 |
|  | miRLet7i | Cor.Coeff. | -.616^**^ | -.459^**^ | -.455^**^ | .328^**^ | .389^**^ | .507^**^ | 0.98** | .505^**^ | .379^**^ | .413^**^ |
|  |  | Sig. (2-tailed) | .< 0.01 | .< 0.01 | .< 0.01 | .001 | .< 0.01 | .< 0.01 | 0.00. | .< 0.01 | .< 0.01 | .< 0.01 |
|  | Serum TLR1 | Cor.Coeff. | **-.615^**^** | **-.503^**^** | **-.666^**^** | **.428^**^** | **.359^**^** | **.534^**^** | **.505^**^** | 0.99** | .655^**^ | .626^**^ |
|  |  | Sig. (2-tailed) | .< 0.01 | .< 0.01 | .< 0.01 | .< 0.01 | .< 0.01 | .< 0.01 | .< 0.01 | 0.00. | .< 0.01 | .< 0.01 |
|  | Serum TLR7 | Cor.Coeff. | **-.505^**^** | **-.457^**^** | **-.527^**^** | **.400^**^** | **.309^**^** | **.497^**^** | **.379^**^** | .655^**^ | 0.96** | .559^**^ |
|  |  | Sig. (2-tailed) | .< 0.01 | .< 0.01 | .< 0.01 | .< 0.01 | .002 | .< 0.01 | .< 0.01 | .< 0.01 | 0.00. | .< 0.01 |
|  | Serum TLR8 | Cor.Coeff. | **-.521^**^** | **-.458^**^** | **-.507^**^** | **.416^**^** | **.464^**^** | **.501^**^** | **.413^**^** | .626^**^ | .559^**^ | 0.98** |
|  |  | Sig. (2-tailed) | .< 0.01 | .< 0.01 | .< 0.01 | .< 0.01 | .< 0.01 | .< 0.01 | .< 0.01 | .< 0.01 | .< 0.01 | 0.00. |
| **. Correlation is significant at the 0.01 level (2-tailed). | | | | | | | | | | | | |
| *. Correlation is significant at the 0.05 level (2-tailed). | | | | | | | | | | | | |

- Bioinformatics Analysis performed in this study:

1. TLR retrieval:

TLR-CRC disease expression:

In this study we have retrieved three TLRs from databases based on their gene expression in CRC. These TLRs are TLR1, TLR7 and TLR8.


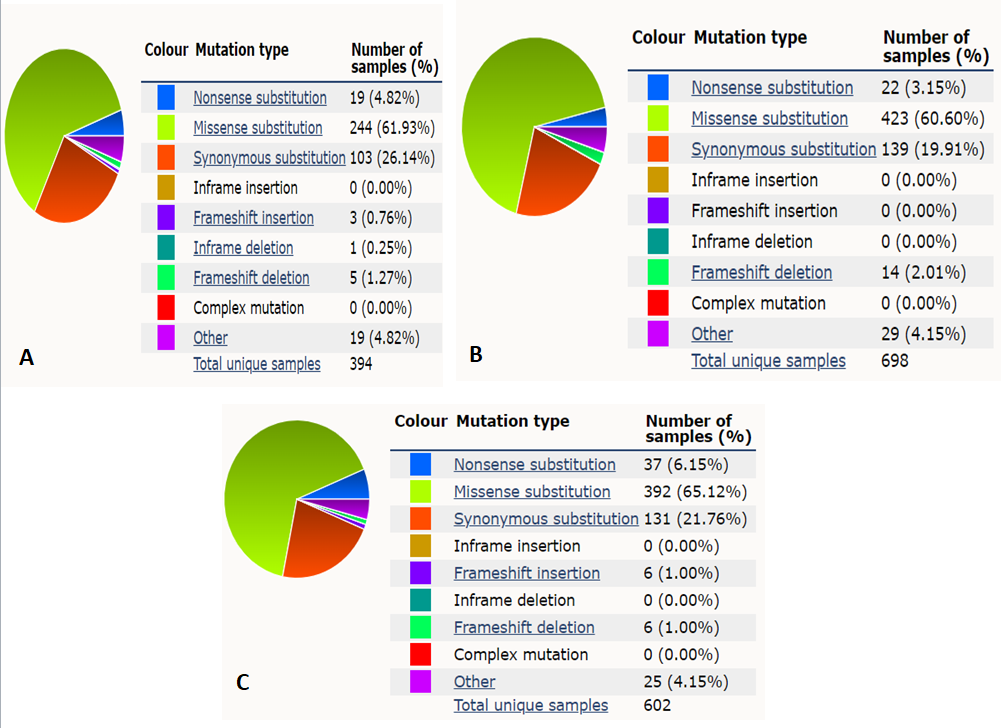


**Figure S1** Chart that shows the distribution of different types of mutations among different cancer types for TLR1(<https://cancer.sanger.ac.uk/cosmic/gene/analysis?ln=TLR1> ), TLR7(<https://cancer.sanger.ac.uk/cosmic/gene/analysis?ln=TLR7> ) and <https://cancer.sanger.ac.uk/cosmic/gene/analysis?ln=TLR8_ENST00000218032>

1.1 TLR1:


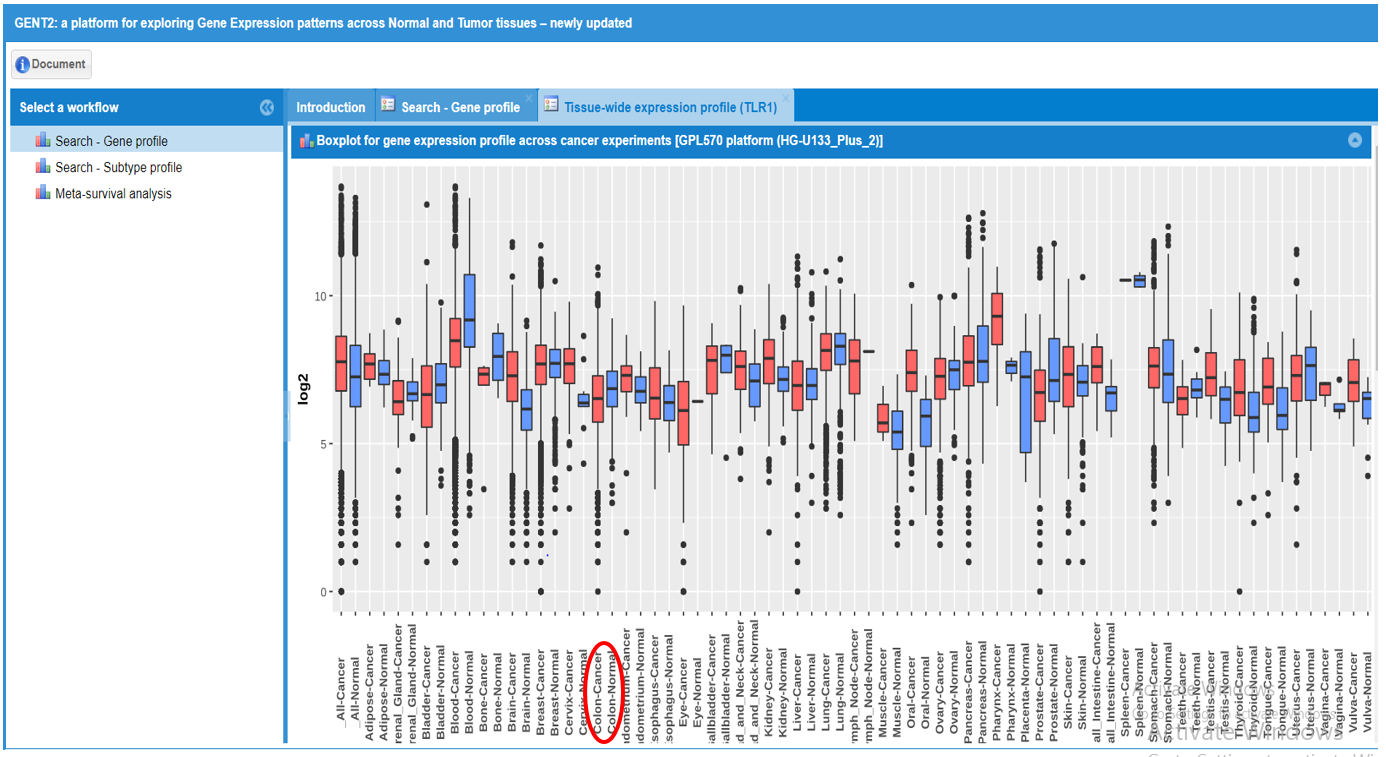


**Figure S2 :**Screenshot from updated GENT2 database at GPL570 platform (HG-U133_Plus_2) dataset showing TLR1 differential gene expression in normal colon tissue and colon cancer tissue with p value<0.001 and Log2FC=-0.309.

**Available at:** <http://gent2.appex.kr/gent2/> , **Accessed on:** 18/9/2022

1.2 TLR7:


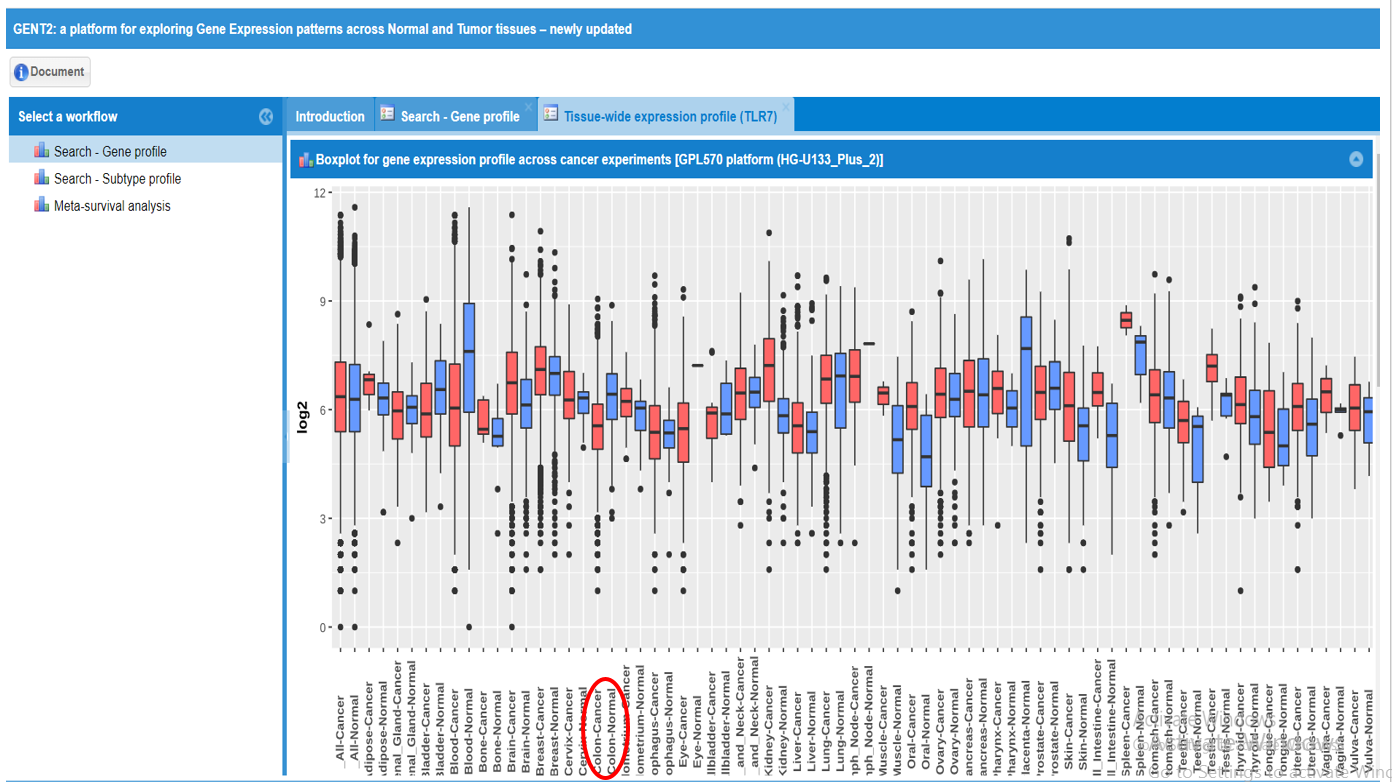


**Figure S3 :**Screenshot from updated GENT2 database at GPL570 platform (HG-U133_Plus_2) dataset showing TLR7 differential gene expression in normal colon tissue and colon cancer tissue with p value<0.001 and Log2FC=-0.806.

**Available at:** <http://gent2.appex.kr/gent2/> , **Accessed on:** 18/9/2022

1.3 TLR8:


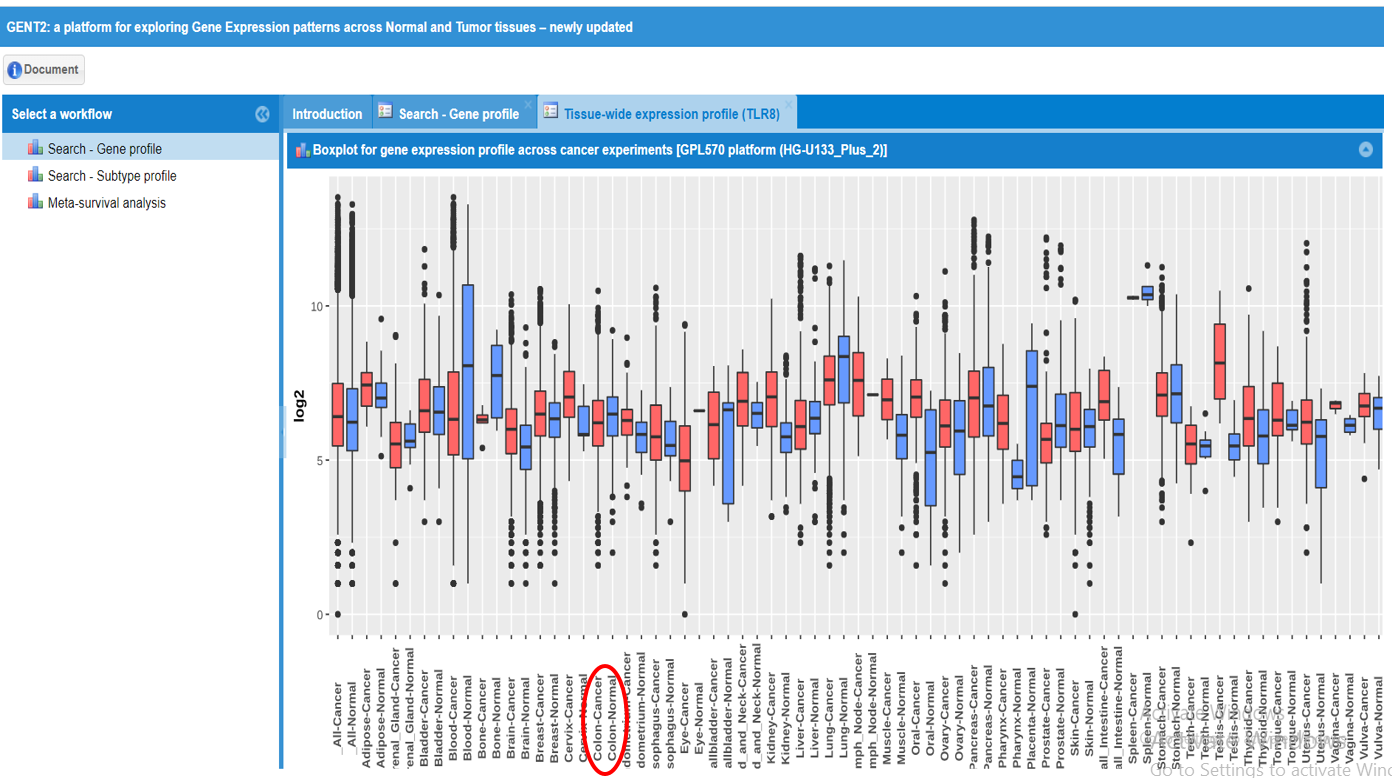


**Figure S4 :**Screenshot from updated GENT2 database at GPL570 platform (HG-U133_Plus_2) dataset showing TLR8 differential gene expression in normal colon tissue and colon cancer tissue with p value=0.004 and Log2FC=-0.157

**Available at:** <http://gent2.appex.kr/gent2/> , **Accessed on:** 18/9/2022

- We have also verified our selection form gene expression atlas database that displays different experiments with different biological conditions for TLR1,7,8 in colorectal cancer in homosapiens, however the strong blue color in the following figures is an evidence of their upregulation:


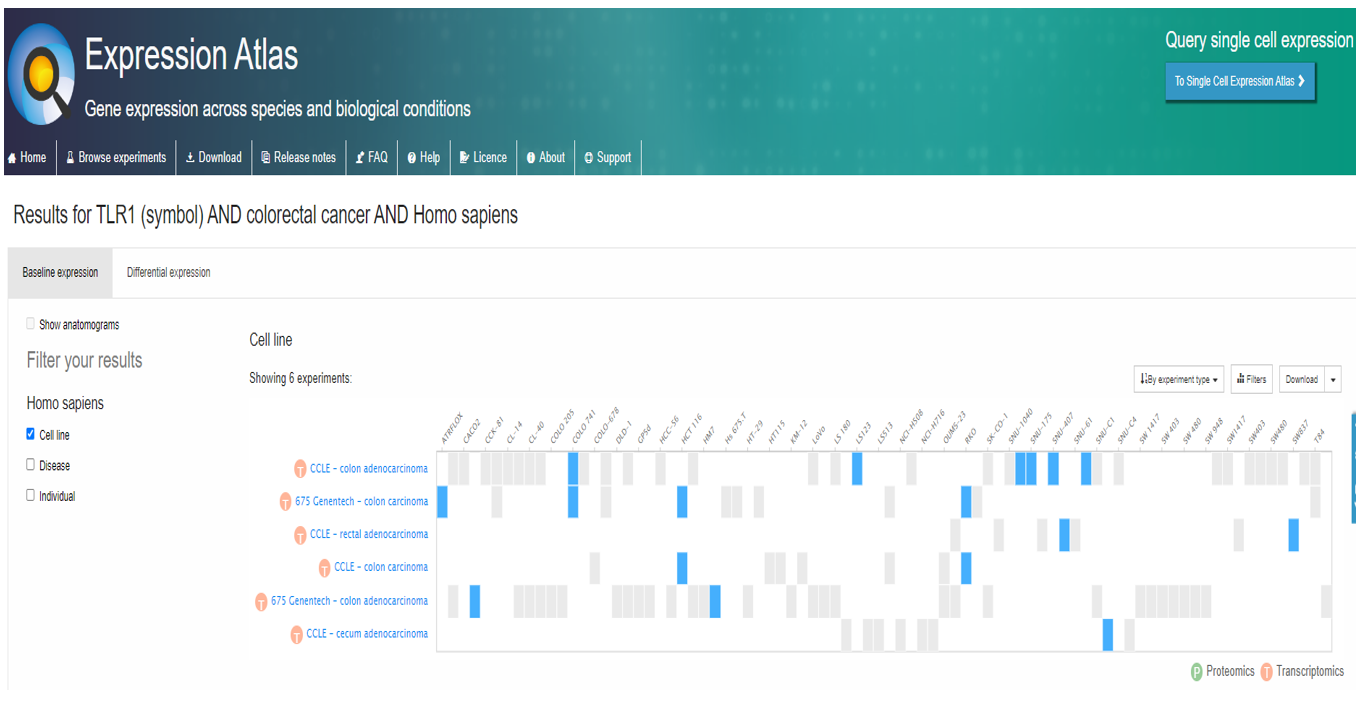


**Figure S5 :**Screenshot from Gene Expression atlas database showing TLR1 differential gene expression in different experiments of colorectal cancer.

**Available at:** <https://www.ebi.ac.uk/gxa/home> , **Accessed on:** 18/9/2022


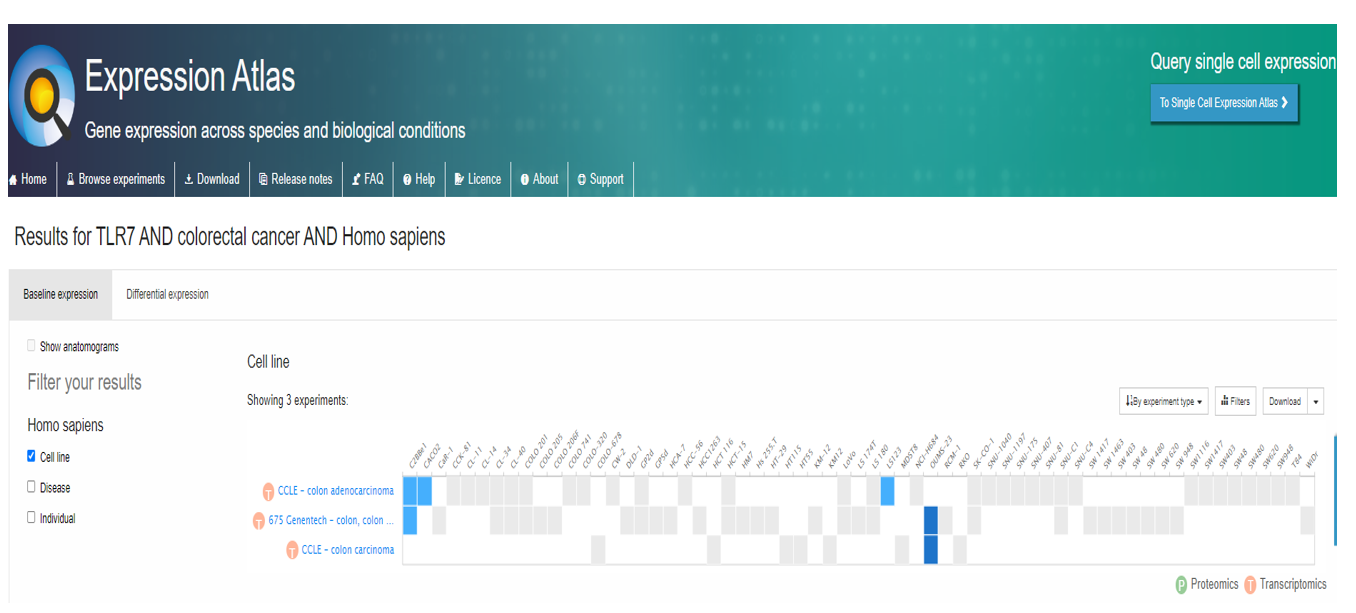


**Figure S6 :**Screenshot from Gene Expression atlas database showing TLR7 differential gene expression in different experiments of colorectal cancer.

**Available at:** <https://www.ebi.ac.uk/gxa/home> , **Accessed on:** 18/9/2022


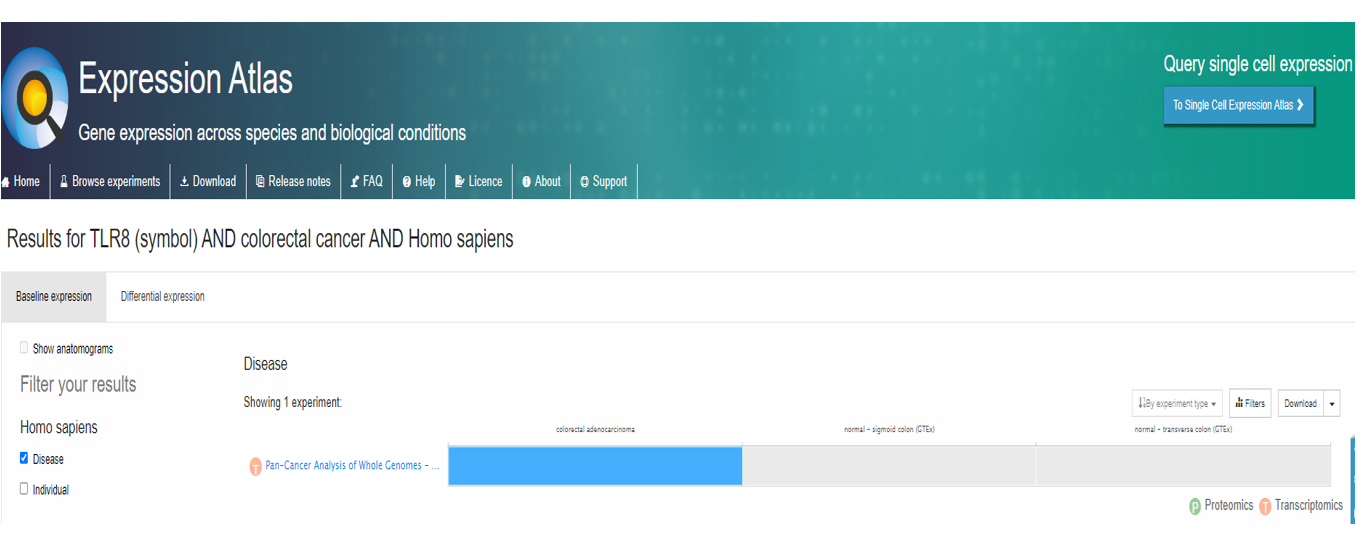


**Figure S7 :**Screenshot from Gene Expression atlas database showing TLR8 differential gene expression in different experiments of colorectal cancer.

**Available at:** <https://www.ebi.ac.uk/gxa/home> , **Accessed on:** 18/9/2022

2. TLR-gene ontology:


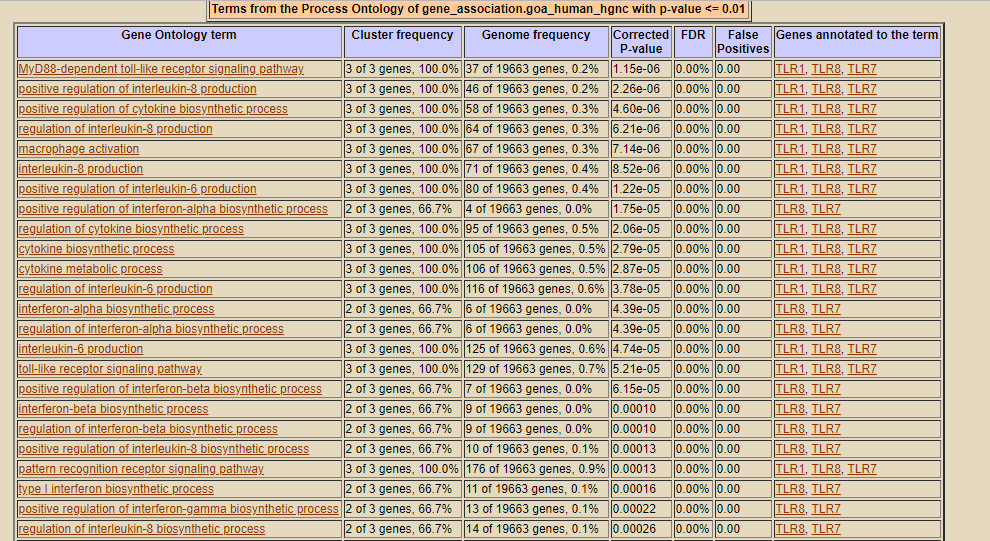


**Figure S8:**Screenshot from Princeton Gene ontology tools database : TLR1 ,TLR7 and TLR8 genes are implicated in cancer and various immunomodulatory pathways.

Available at: <https://go.princeton.edu/cgi-bin/GOTermFinder>

3. miRNA retrieval suspected to act as TLR ligand:

miRNA-CRC disease expression:

In this study we have retrieved seven miRNAs from databases based on their gene expression in CRC. These miRNAs are miRNA 202, miRNA let7i , miRNA 122, miRNA98, miRNA 29b, miRNA21, miRNA15b.

3.1 miRNA -202:


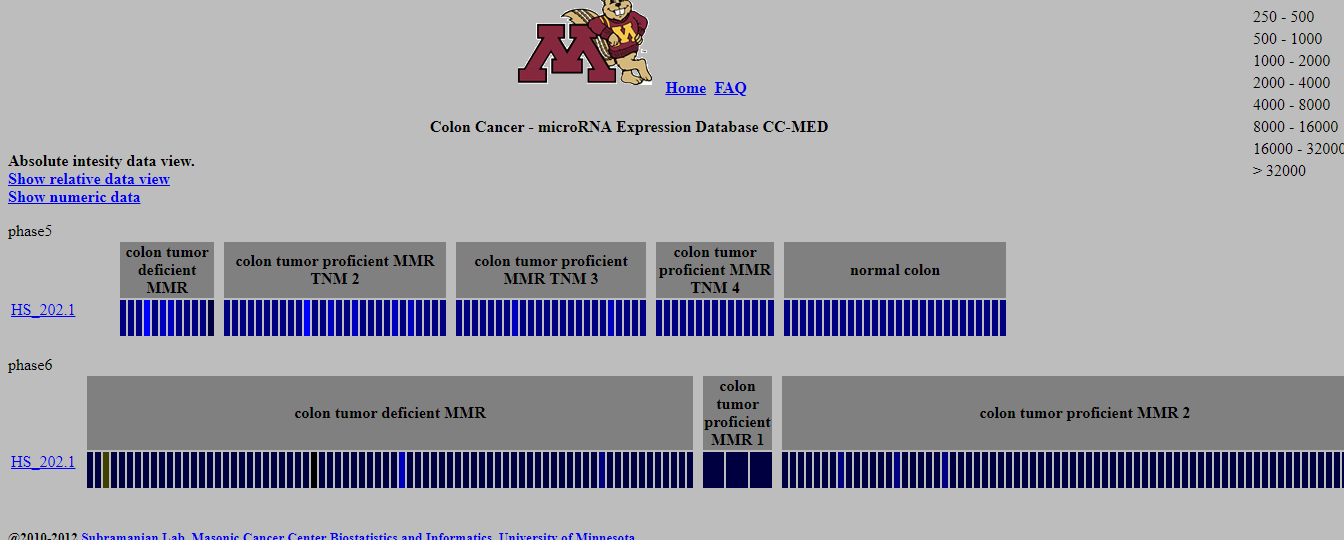


**Figure S9 :**Screenshot from Colon cancer miRNA expression database: miRNA 202expression in different stages of colon cancer tissue in human.

Available at:[https](file:///C:\Users\Nourhan\Desktop\paper2021\https)[://www.oncomir.umn.edu/colon/basic_search.php](https://www.oncomir.umn.edu/colon/basic_search.php)

3.2 miRNA let7i:


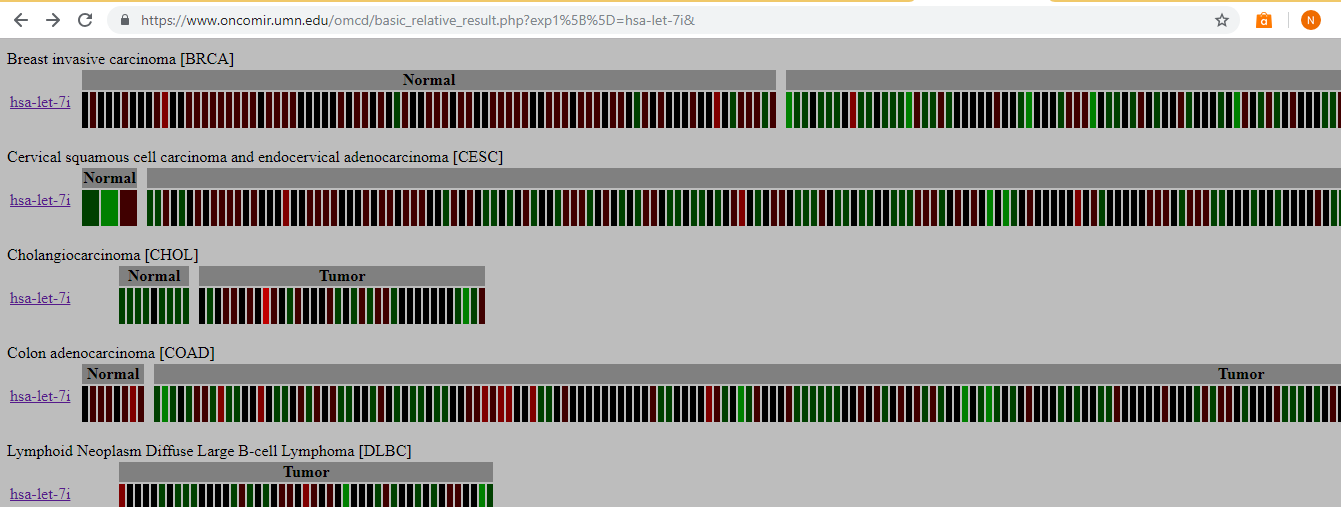
**Figure S10 :**Screenshot from Colon cancer miRNA expression database: miRNA let7i expression in different stages of colon cancer tissue in human. Available at:[https](file:///C:\Users\Nourhan\Desktop\paper2021\https)[://www.oncomir.umn.edu/colon/basic_search.php](https://www.oncomir.umn.edu/colon/basic_search.php)

3.3 miRNA 122:


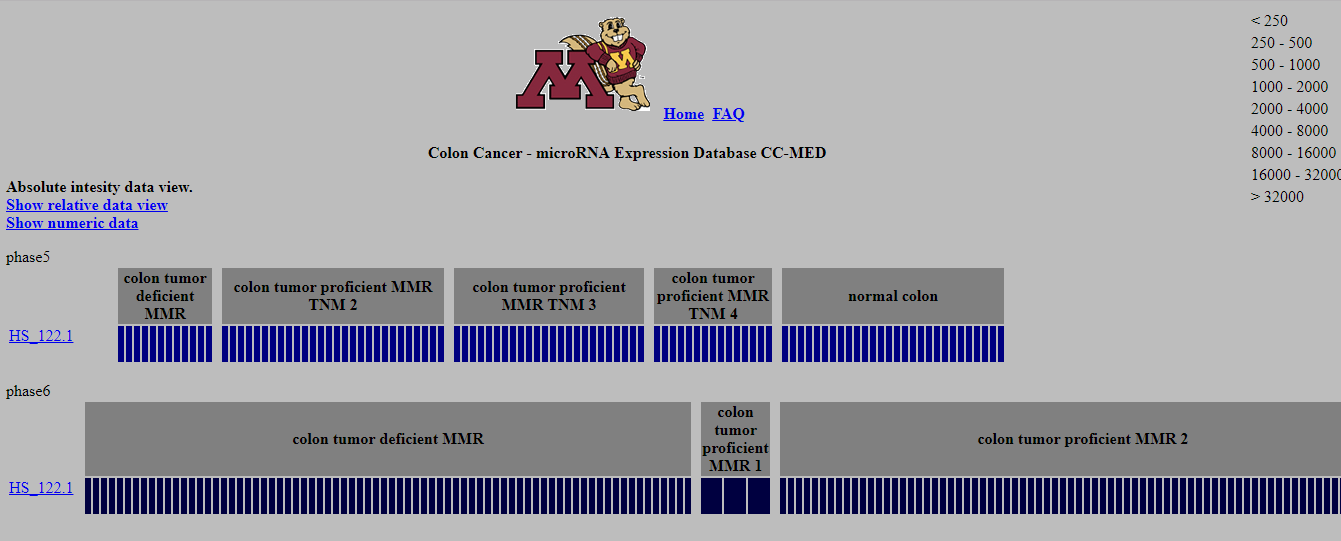


**Figure S11** :Screenshot from Colon cancer miRNA expression database: miRNA 122expression in different stages of colon cancer tissue in human.

Available at:[https](file:///C:\Users\Nourhan\Desktop\paper2021\https)[://www.oncomir.umn.edu/colon/basic_search.php](https://www.oncomir.umn.edu/colon/basic_search.php)

3.4 miRNA 98:


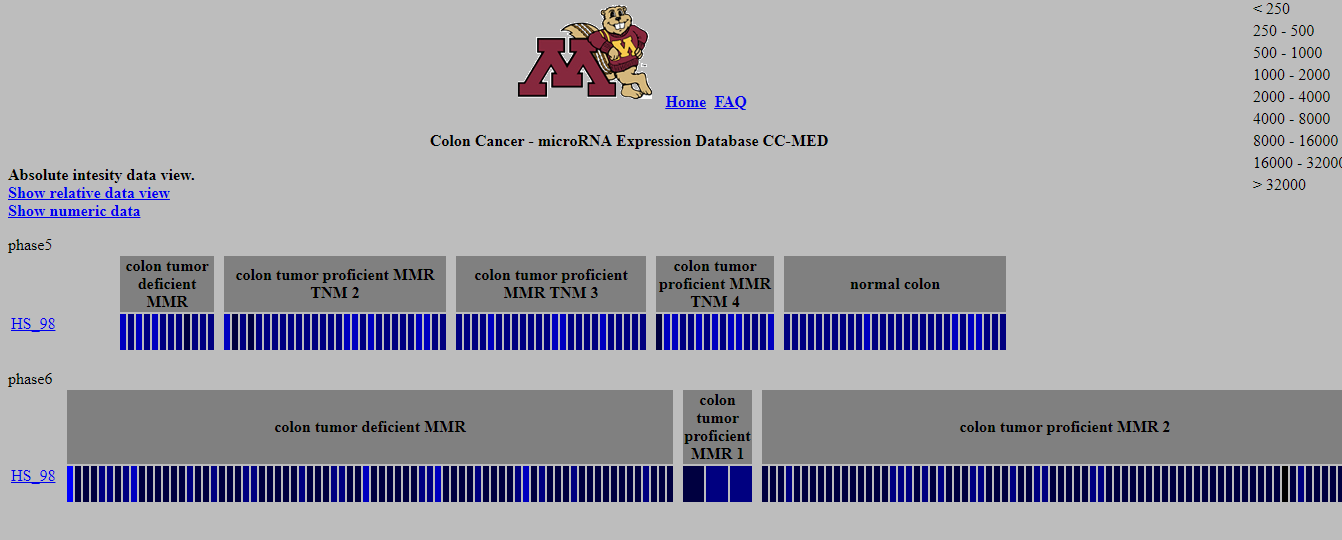


**Figure S12** :Screenshot from Colon cancer miRNA expression database: miRNA 98 expression in different stages of colon cancer tissue in human.

Available at:[https](file:///C:\Users\Nourhan\Desktop\paper2021\https)[://www.oncomir.umn.edu/colon/basic_search.php](https://www.oncomir.umn.edu/colon/basic_search.php)

3.5 miRNA 29b:


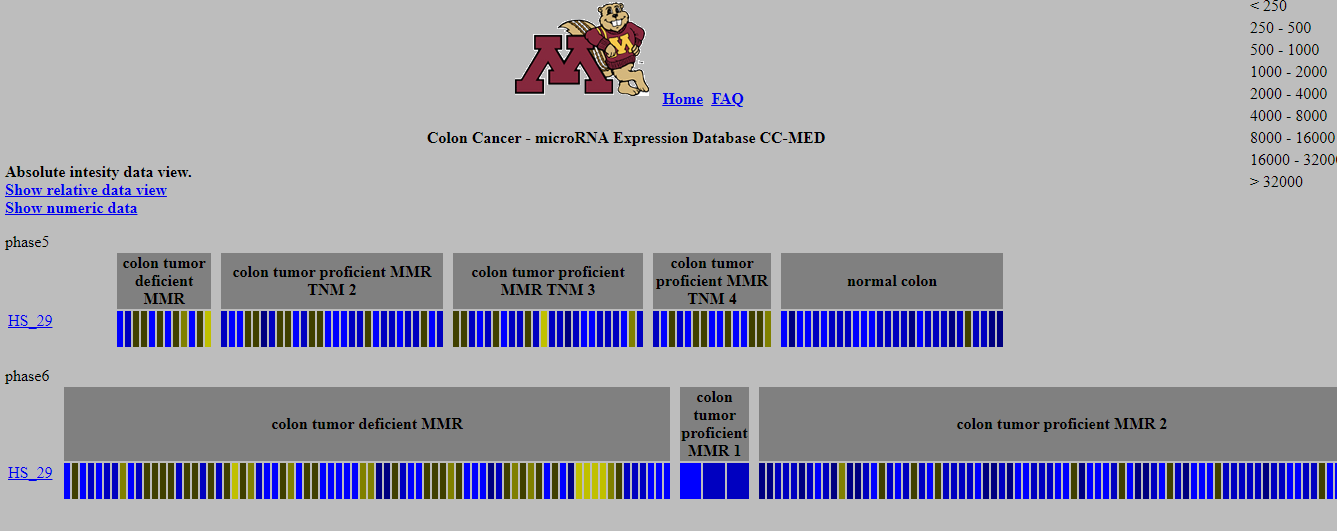


**Figure S13** :Screenshot from Colon cancer miRNA expression database: miRNA 29b expression in different stages of colon cancer tissue in human.

Available at:[https](file:///C:\Users\Nourhan\Desktop\paper2021\https)[://www.oncomir.umn.edu/colon/basic_search.php](https://www.oncomir.umn.edu/colon/basic_search.php)

3.6 miRNA 21:


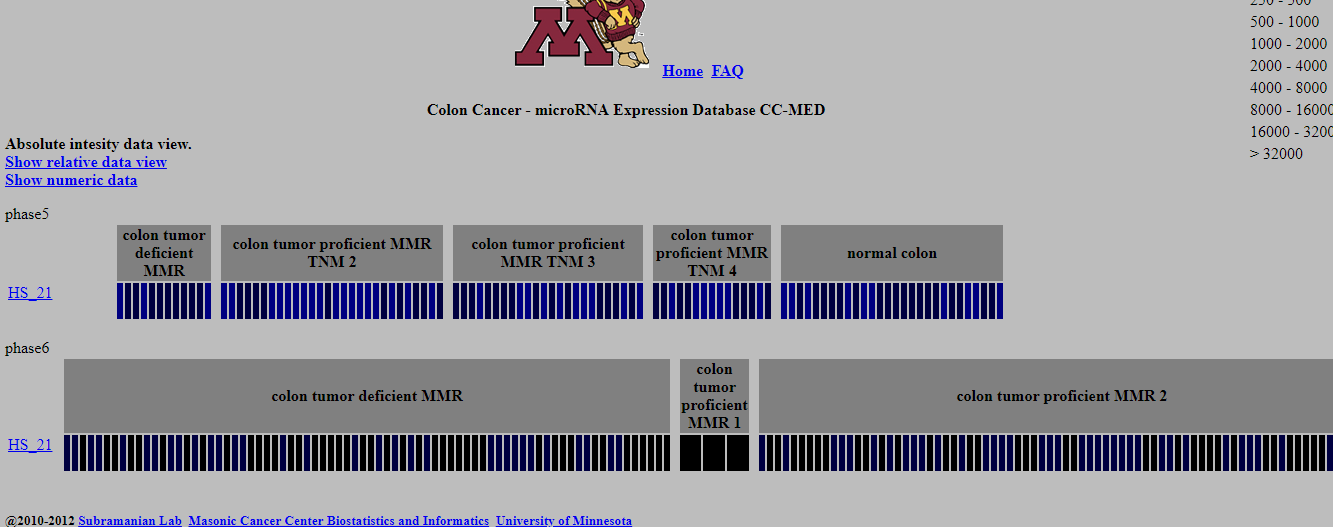


**Figure S14** :Screenshot from Colon cancer miRNA expression database: miRNA 21 expression in different stages of colon cancer tissue in human. Available at:[https](file:///C:\Users\Nourhan\Desktop\paper2021\https)://www.oncomir.umn.edu/colon/basic_search.php


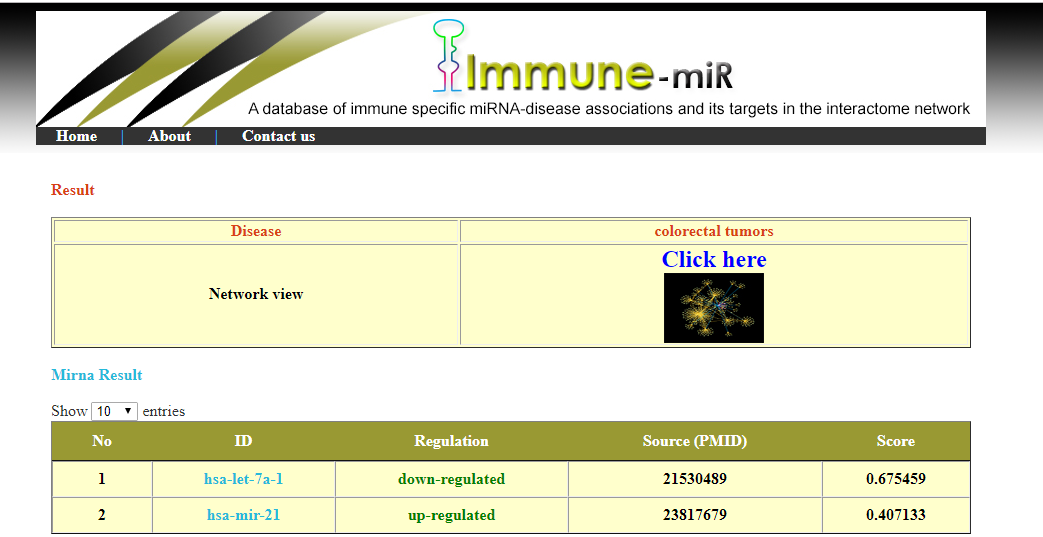


**Figure S15 :**Screenshot from Immune-miR database: miRNA 21 expression in colon cancer tissue in human. Available at:<http://biominingbu.org/immunemir/index.html>

3.7 miRNA 15b:


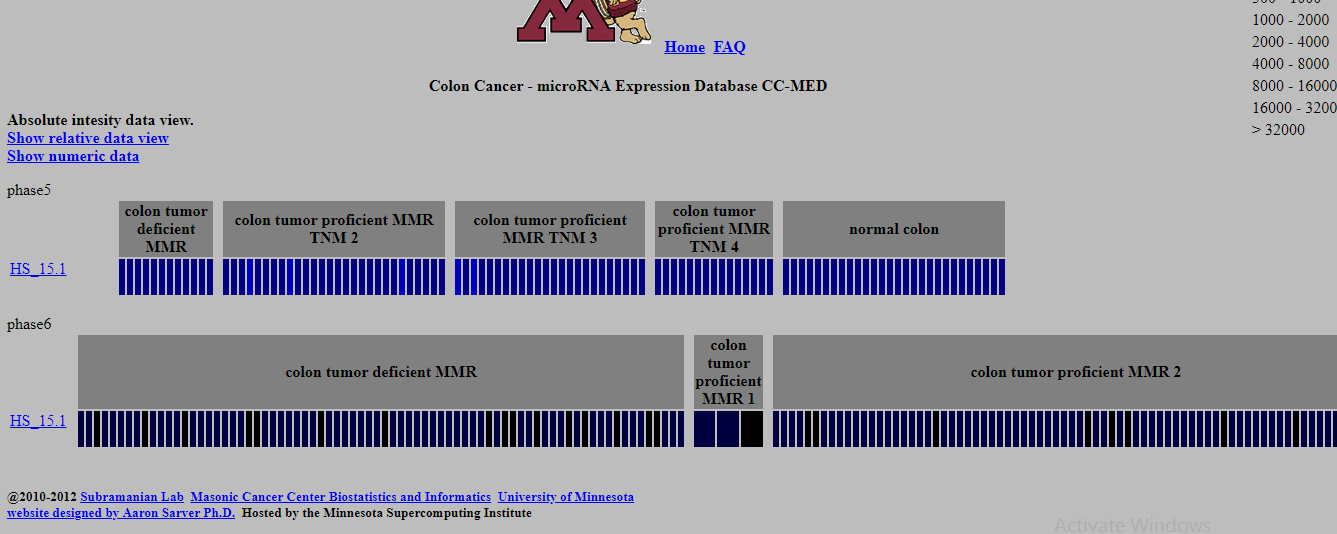


**Figure S16** :Screenshot from Colon cancer miRNA expression database: miRNA 15b expression in different stages of colon cancer tissue in human.

Available at:[https](file:///C:\Users\Nourhan\Desktop\paper2021\https)[://www.oncomir.umn.edu/colon/basic_search.php](https://www.oncomir.umn.edu/colon/basic_search.php)

4. miRNA-pathway enrichment analysis:


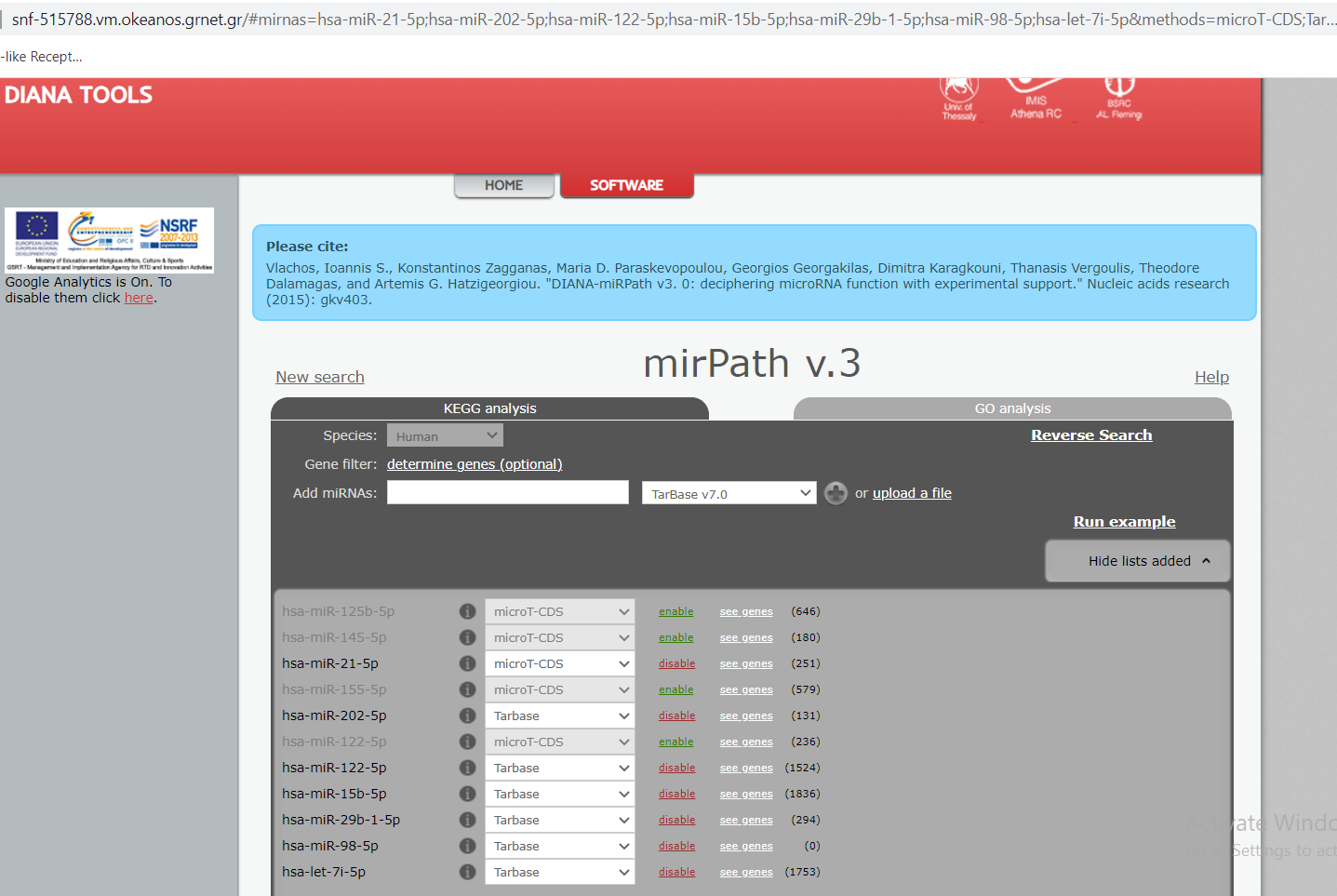


A


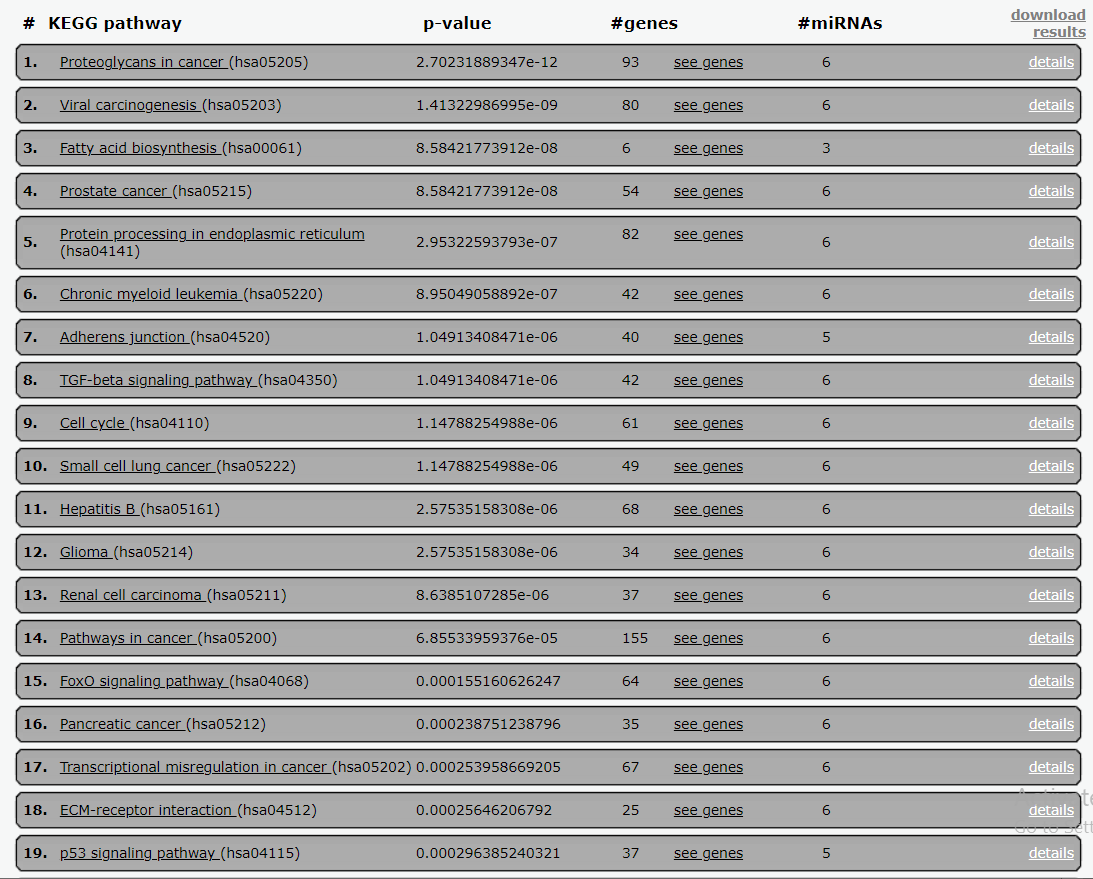
 **Figure S17 (A,B)** :Screenshot from **DIANA tools-miRpath V.3**: The selected miRNAs miRNA 202, miRNA let7i , miRNA 122, miRNA98, miRNA 29b, miRNA21, miRNA15b are implicated in cancer and various immunomodulatory pathways.

B

Available at:<http://snf-515788.vm.okeanos.grnet.gr/index.php?r=mirpath>

5. Docking of selected TLRs and miRNAs:

**Docking Methodology:**

Protein-protein docking was performed to miRNAs in TLRs using ZDOCK protocol, ZDOCK is a protein-protein docking algorithm that applies ligand rotation and a pair-wise shape complementarity method that takes advantage of fast Fourier transformation. All chains and receptors chosen showed 100% similarity through sequence search on the Protein Data Bank and NCBI sequence and fasta search.

**PDB codes of TLRs used were as follows:**

- TLR1 (pdb code 6NIH, crystal structure of human TLR1
- **^^[[1]](#endnote-1)^^**)
- TLR7 (pdb code 5GMH, crystal structure of monkey TLR7 in complex with R848**^^[[2]](#endnote-2)^^**)
- TLR8 (pdb code 3WN4, crystal structure of human TLR8 in complex with DS-877**^^[[3]](#endnote-3)^^**)

**microRNAs used were as follows:**

- miRNA-122 (Chain C in pdb code 6MDZ, Human Argonaute2-miR-122 bound to a target RNA with two centra; mismatches “bu2”**^^[[4]](#endnote-4)^^**)
- miRNA-21 (Chain A in pdb code 5UZT, structure of wild type pre-miR21 apical loop**^^[[5]](#endnote-5)^^**)
- miRNA-202 (pdb code 6N5O, structure of human pir-miRNA apical loop and one-base-pair fused to the YdaO riboswitch scaffold)
- miRNA-15b (Chain A in pdb code 4UE4, structural basis for targeting and elongation arrest of Bacillus signal recognition particle**^^[[6]](#endnote-6)^^**)
- miRNA-29b (Chain F in pdb code 5Y88, Cryo-EM structure of the intron-lariat splicesome ready for disassembly from S. cerevisiae at 3.5 angstrom**^^[[7]](#endnote-7)^^**)

**Docking runs performed were as follows:**

1. miRNA-21, miRNA-122 and miRNA-15b were docked in TLR1

2. miRNA-21 and miRNA-202 were docked in TLR7

3. miRNA-21 and miRNA-29b were docked in TLR8

**Docking Results:**


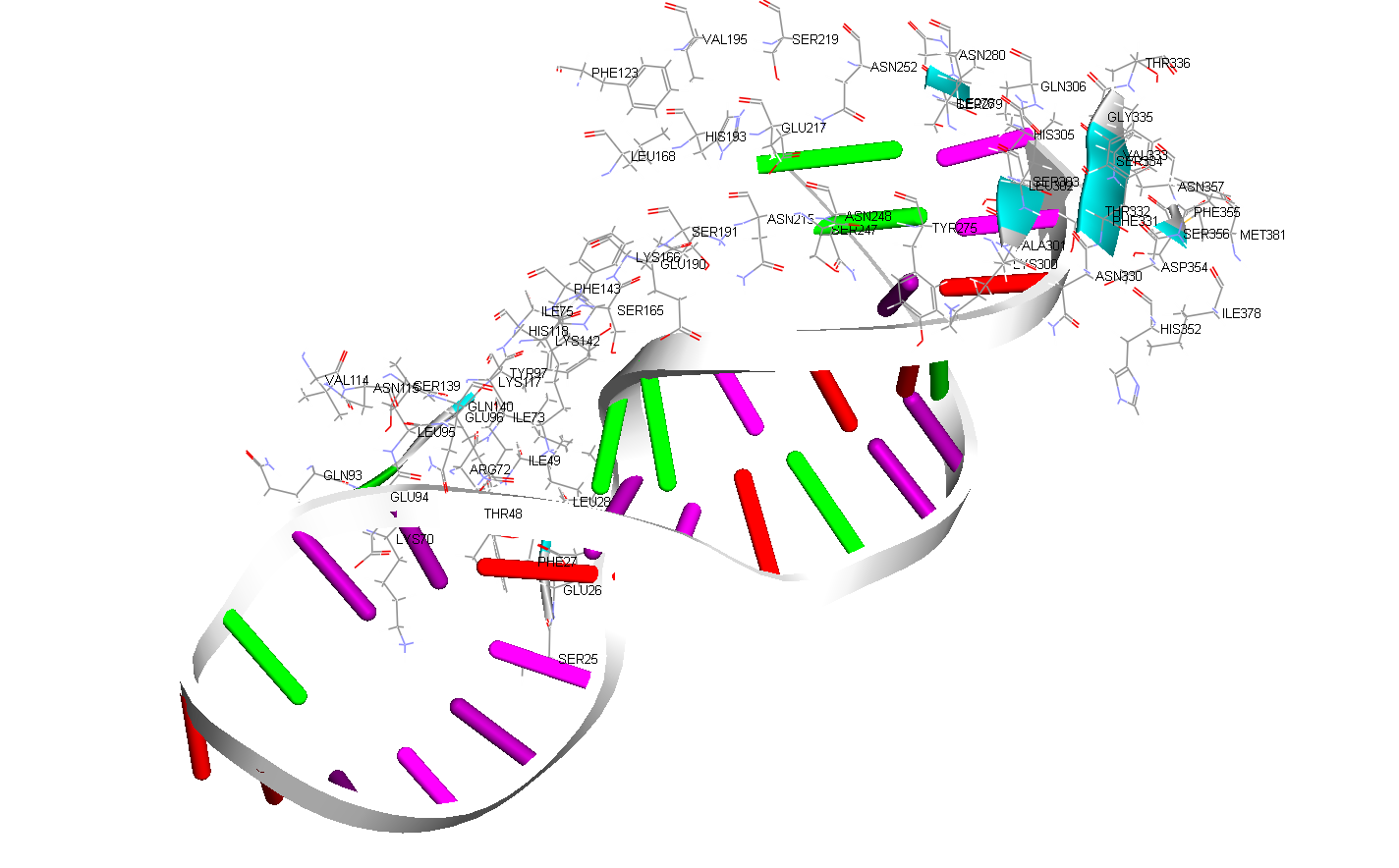


**Figure S18:** Docking of miRNA-21 into TLR1 insilico with ZDock score 22.9.

**Amino acids in the binding interface:**

Ser25, Glu26, Phe27, Leu28, Thr48, Ile49, Lys70, Arg72, Ile73, Ile75, Gln93, Glu94, Leu95, Glu96, Tyr97, Val114, Asn115, Lys117, His118, Phe123, Ser139, Gln140, Lys142, Phe143, Ser165, Lys166, Leu168, Glu190, Ser191, His193, Val195, Asn215, Glu217, Ser219, Ser247, Asn248, Asn252, Tyr275, Ile278, Ser279, Asn280, Lys300, Ala301, Leu302, Ser303, His305, Gln306, Asn330, Phe331, Thr332, Val333, Ser334, Gly335, Thr336, His352, Asp354, Phe355, Ser356, Asn357, Ile378, Met381.


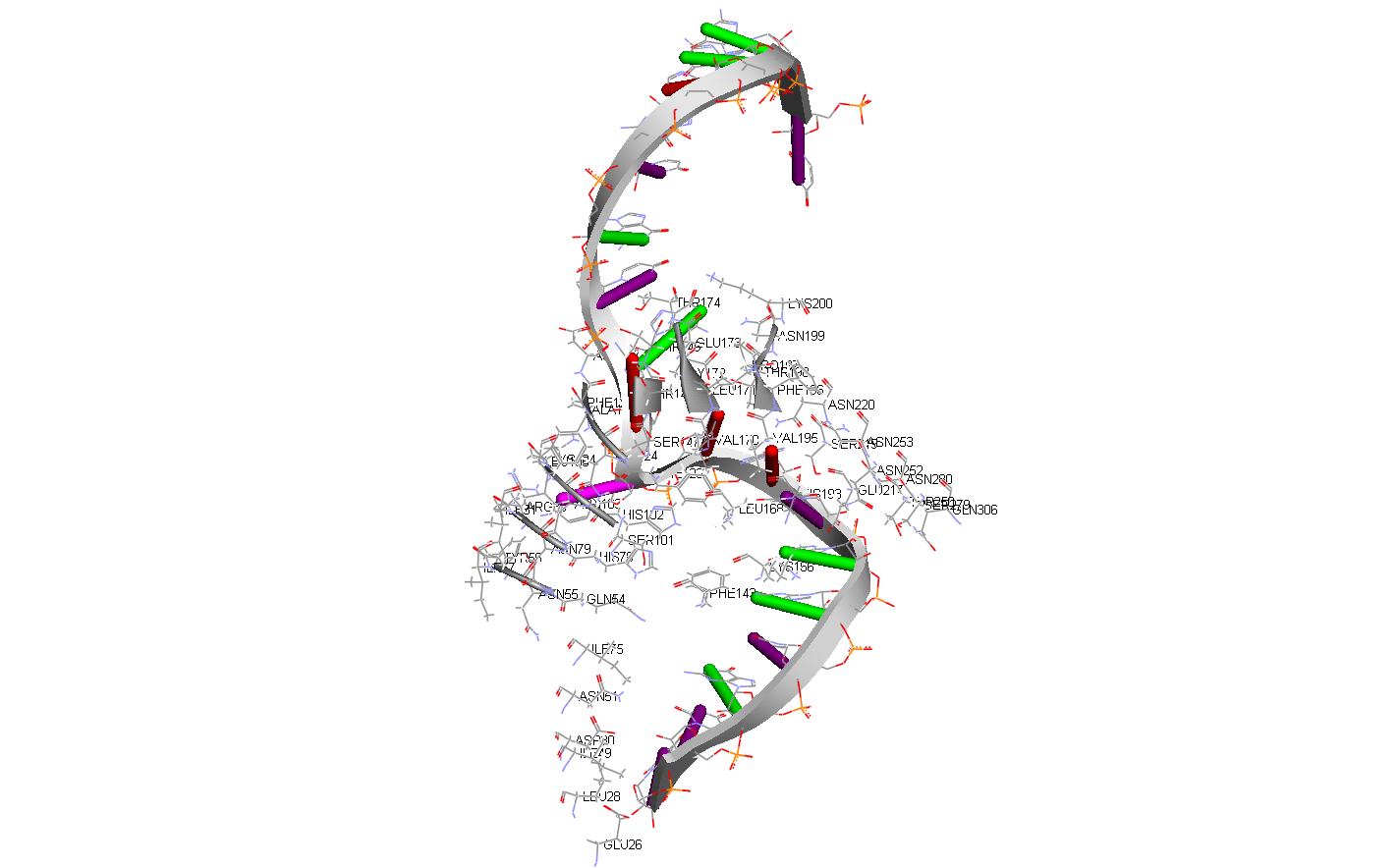


**Figure S19:** Docking of miRNA-122 into TLR1 insilico with ZDock score 20.56.

**Amino acids in the binding interface:**

Glu26, Leu28, Asp30, Ile49, Asn51, Gln54, Asn55, Tyr56, Ile57, Ile75, His78, Asn79, Arg80, Ile81, Ser101, His102, Asn103, Lys104, Leu105, Phe123, Asn124, Ala125, Phe126, Asp127, Phe143, Ser147, Thr148, Thr149, Lys166, Leu168, Val170, Leu171, Gly172, Glu173, Thr174, His193, Val195, Phe196, Pro197, Thr198, Asn199, Lys200, Glu217, Ser219, Asn220, Thr250, Asn252, Asn253, SEr279, Asn280, Gln306.


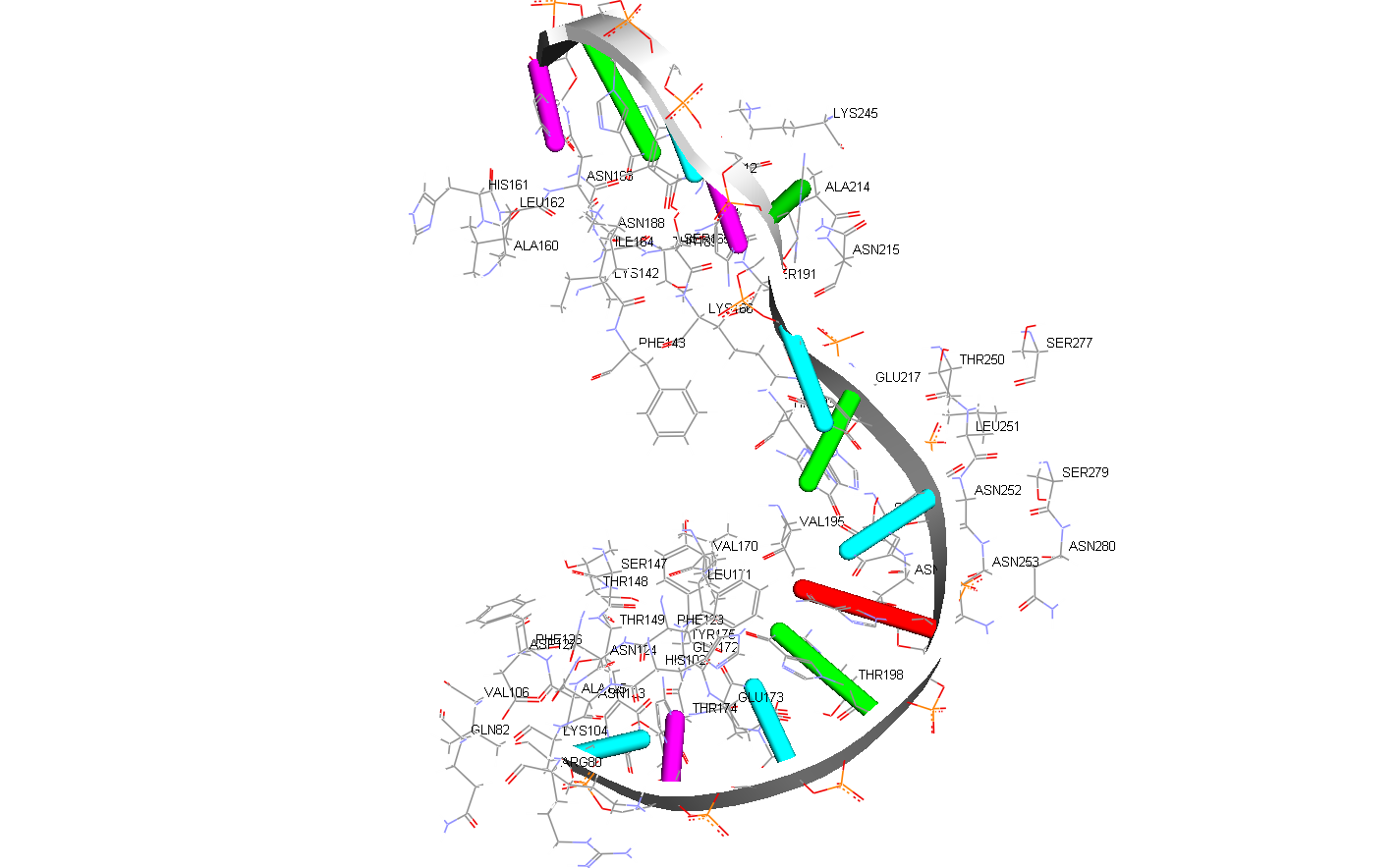


**Figure S20:** Docking of miRNA-15b into TLR1 insilico with ZDock score 21.66.

**Amino acids in the binding interface:**

Arg80, Gln82, His102, Asn103, Lys104, Val106, Phe123, Asn124, Ala125, Phe126, Asp127, Lys142, Phe143, Ser147, Thr148, Thr149, Ala160, His161, Leu162, Asn163, Ile164, Ser165, Lys166, Val170, Leu171, Gly172, Glu173, Thr174, Tyr175, Asn188, Thr189, Glu190, Ser191, His193, Val195, Thr198, Thr212, Ala214, Asn215, Glu217, Ser219, Asn220, Lys245, Thr250, Leu251, Asn252, Asn253, Ser277, Ser279, Asn280.


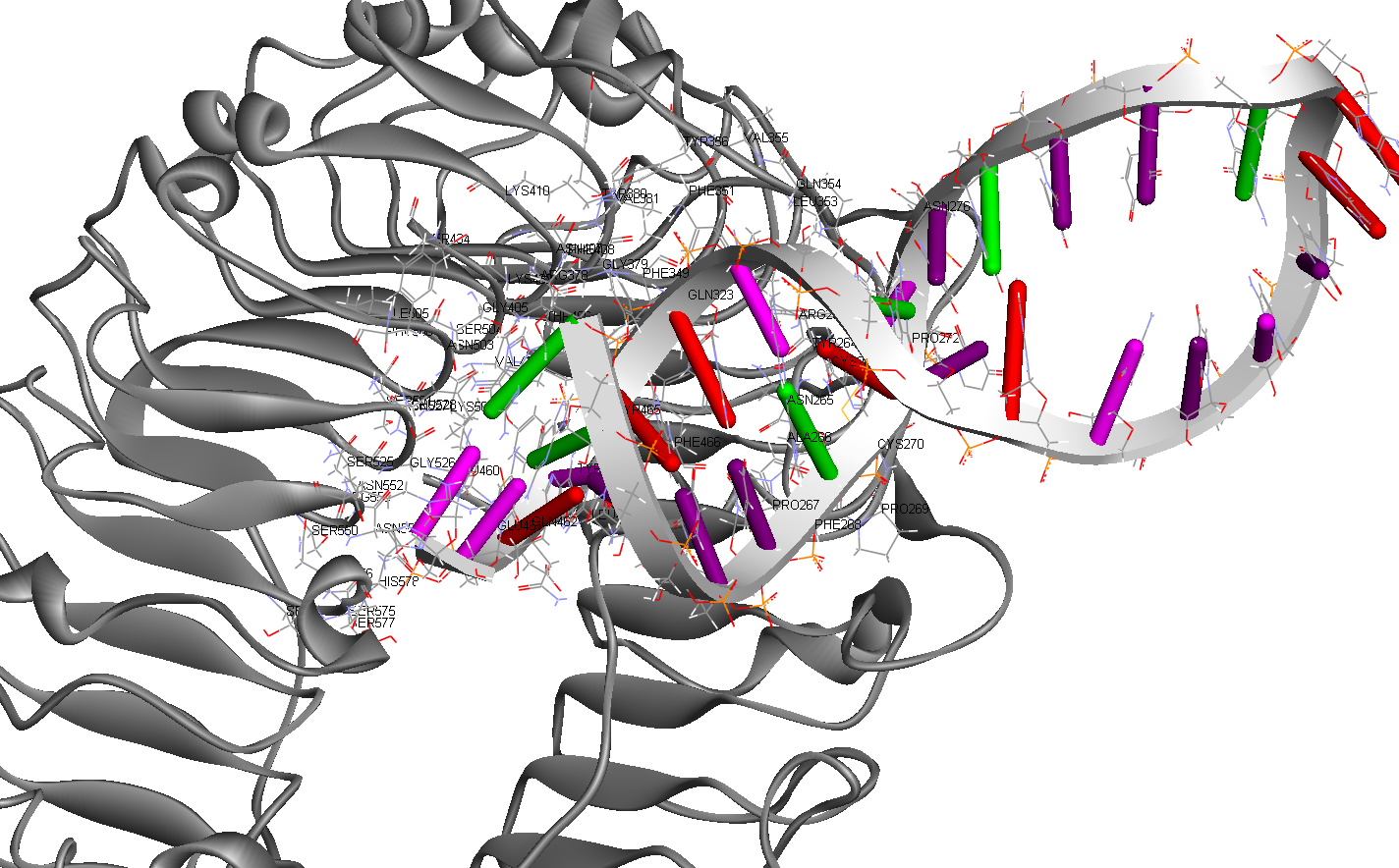


**Figure S21:** Docking of miRNA-21 into TLR7 insilico with ZDock score 25.12.

**Amino acids in the binding interface:**

Arg262, Cys263, Tyr264, Asn265, Ala266, Pro267, Phe268, Pro269, Cys270, Pro272, Asn276, Gln323, Phe349, Phe351, Leu353, Gln354, Val355, Tyr356, Arg378, Gly379, Tyr380, Val381, Gly405, Thr406, Asn407, Phe408, Lys410, Val430, Lys432, Ser434, Leu460, Glu461, Gln462, Leu463, Tyr464, Tyr465, Phe466, Ser501, Lys502, Asn503, Ser504, Ile505, Phe506, Ser525, Gly526, Asn527, Leu528, Ser550, Asn551, Asn552, Arg553, Ser574, Ser575, Asn576, Ser577, His578.


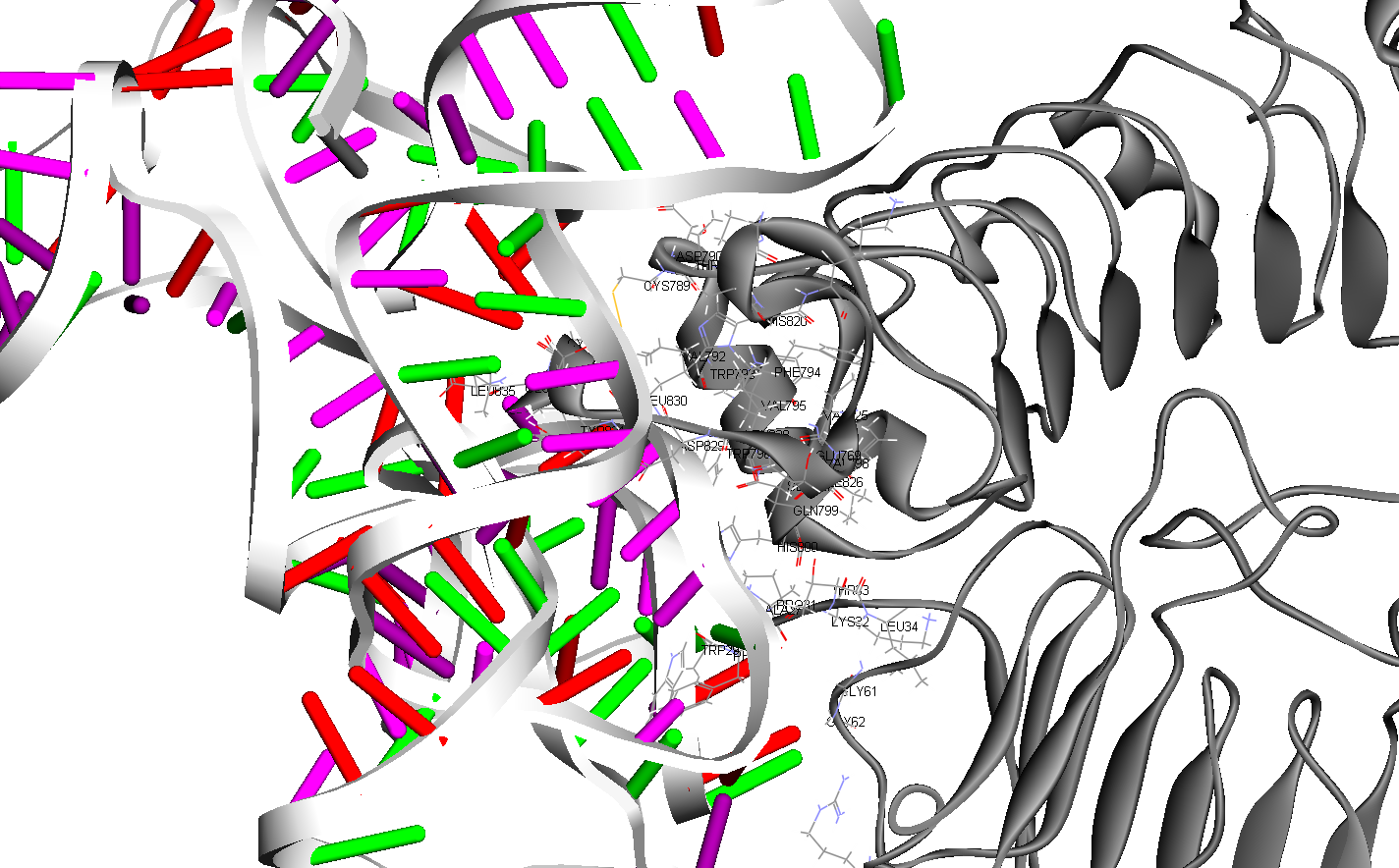


**Figure S22:** Docking of miRNA-202 into TLR7 insilico with ZDock score 25.96.

**Amino acids in the binding interface:**

Ala27, Trp29, Phe30, Pro31, Lys32, Thr33, Leu34, Gly61, Gly62, Arg87, Glu769, Thr788, Cys789, Asp790, Val792, Trp793, Phe794, Val795, Trp796, Trp797, Val798, Gln799, His800, Pro817, Gly818, Ala819, His820, Lys821, Val825, Ile826, Ser827, Leu828, Asp829, Leu830, Tyr831, Thr832, Cys833, Glu834, Leu835


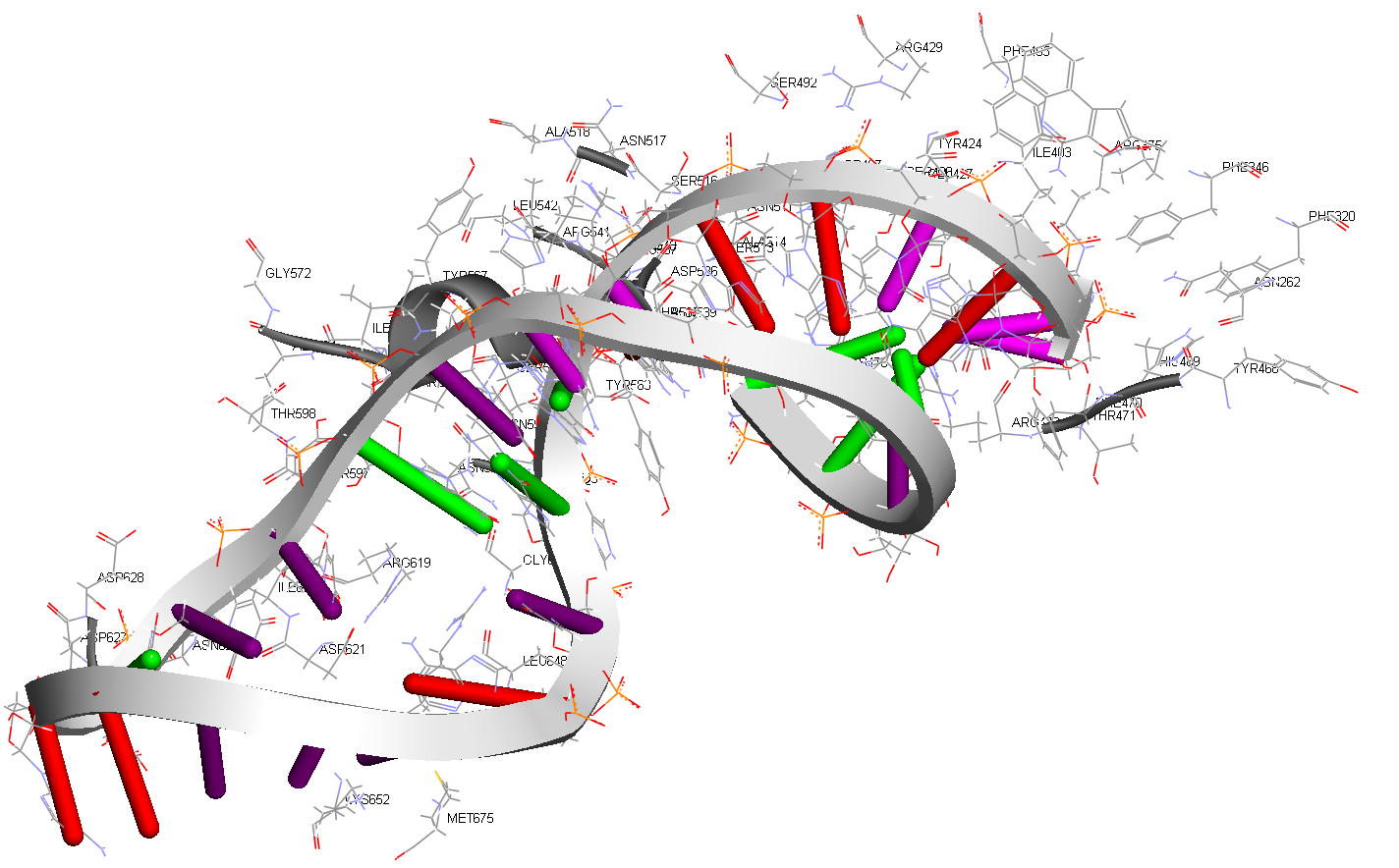


**Figure S23:** Docking of miRNA-21 into TLR8 insilico with ZDock score 25.82.

**Amino acids in the binding interface:**

Asn262, Phe320, Phe346, Arg375, Ile403, Phe405, Tyr424, Ser426, Glu427, Arg429, Tyr468, His469, Phe470, Thr471, Arg472, Lys476, Asp487, Ser489, Leu490, Ser492, Asn511, Ser513, Ala514, Ser516, Asn517, Ala518, Tyr534, Asp536, Thr538, Asn539, Asn540, Arg541, Leu542, Asp560, Tyr563, Asn564, Ser565, His566, Tyr567, Phe568, Arg569, Ile570, Ala571, Gly572, Ser592, His593, Asn594, Asn595, Tyr597, Thr598, Gly617, Arg619, Asp621, Ile622, Asn625, Asp627, Leu648, Arg650


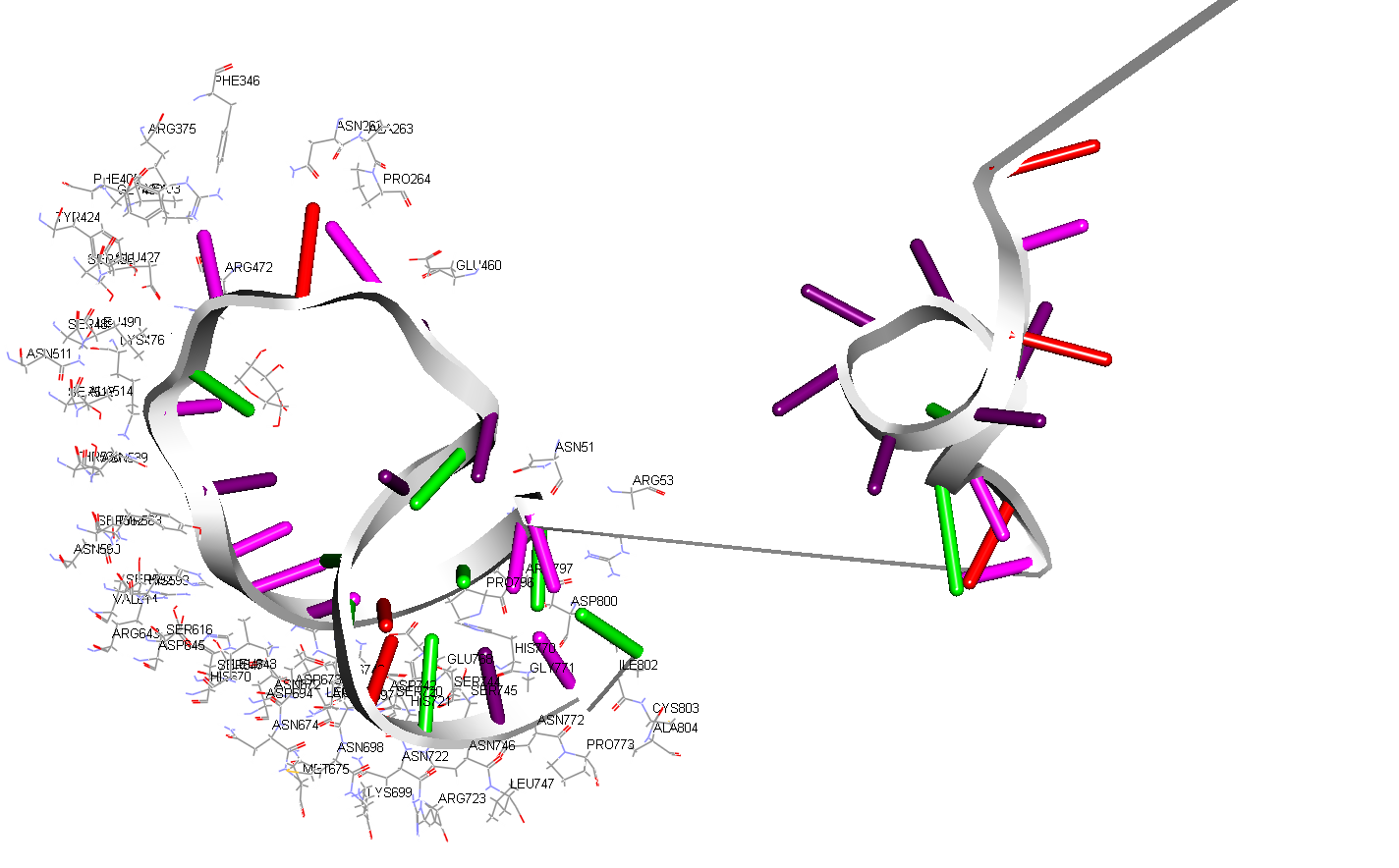


**Figure S24:** Docking of miRNA-29b into TLR8 insilico with ZDock score 29.74.

**Amino acids in the binding interface:**

Asn51, Arg53, Asn262, Ala263, Pro264, Phe346, Arg375, Gly402, Ile403, Phe405, Tyr424, Ser426, Glu427, Glu460, Arg472, Lys476, Ser489, Leu490, Asn511, Ser513, Ala514, Thr538, Asn539, Ser562, Tyr563, Asn590, Ser592, His593, Val614, Ser616, Arg643, Asp645, Ser647, Leu648, His670, Asn672, Asn673, Asn674, Met675, Asp694, Arg696, Gly697, Asn698, Lys699, Leu718, Ser720, His721, Asn722, Arg723, His740, Asp742, Ser744, Ser745, Asn746, Leu747, Glu768, His770, Gly771, Asn772, Pro773, Pro796, Arg797, Asp800, Ile802, Cys803, Ala804

**References**

1. Su L, Wang Y, Wang J, Mifune Y, Morine MD, Jones BT, Moresco EMY, Boger DL, Beutler B, Zhang H. Structural Basis of TLR2/TLR1 Activation by the synthetic agonist Diprovocim. *J. Med. Chem.* 2019, 62, 2938-2949. Doi: [10.1021/acs.jmedchem.8b01583](http://dx.doi.org/10.1021/acs.jmedchem.8b01583) [↑](#endnote-ref-1)
2. Zhang Z, Ohto U, Shibata T, Krayukhina E, Taoka M, Yamauchi Y, Tanji H, Isobe T, Uchiyama S, Miyake K, Shimizu T. Structural Analysis Reveals that Toll-like receptor 7 is a Dual Receptor for Guanosine and Single-Stranded RNA. *Immunity* 2016, 45, 737-748. Doi: [10.1016/j.immuni.2016.09.011](http://dx.doi.org/10.1016/j.immuni.2016.09.011) [↑](#endnote-ref-2)
3. Kokatla HP, Sil D, Tanji H, Ohto U, Malladi SS, Fox LM, Shimizu T, David SA. Structure-Based Design of Novel Human Toll-like Receptor 8 Agonists. *ChemMedChem* 2014, 9(4), 719-723. Doi: [10.1002/cmdc.201300573](http://dx.doi.org/10.1002/cmdc.201300573) [↑](#endnote-ref-3)
4. Sheu-Gruttadauria J, Pawlica P, Klum SM, Wang S, Yario TA, Schirle Oakdale NT, Steitz JA, MacRae IJ. Structural Basis for Target-Directed MicroRNA Degradation. *Mol. Cell* 2019, 75, 1243-1255.e7

   Doi: [10.1016/j.molcel.2019.06.019](http://dx.doi.org/10.1016/j.molcel.2019.06.019) [↑](#endnote-ref-4)
5. Shortridge MD, Walker MJ, Pavelitz T, Chen Y, Yang W, Varani G. A Macrocyclic Peptide Ligand Binds the Oncogenic MicroRNA-21 Precursor and Suppresses Dicer Processing. *ACS Chem. Biol.* 2017, 12, 1611-1620. Doi: [10.1021/acschembio.7b00180](http://dx.doi.org/10.1021/acschembio.7b00180) [↑](#endnote-ref-5)
6. Beckert B, Kedrov A, Sohmen D, Kempf G, Wild K, Sinning I, Stahlberg H, Wilson DN, Beckmann R. Translational Arrest by a Prokaryotic Signal Recognition Particle is Mediated by RNA Interactions. *Nat. Struct. Mol. Biol.* 2015, 22, 767-773.

   Doi: [10.1038/nsmb.3086](http://dx.doi.org/10.1038/nsmb.3086) [↑](#endnote-ref-6)
7. Wan R, Yan C, Bai R, Lei J, Shi Y. Structure of an Intron Lariat Spliceosome from Saccharomyces cerevisiae. *Cell* 2017, 171, 120-132

   Doi: [10.1016/j.cell.2017.08.029](http://dx.doi.org/10.1016/j.cell.2017.08.029) [↑](#endnote-ref-7)
